# Optimized circular RNA vaccines for superior cancer immunotherapy

**DOI:** 10.7150/thno.104698

**Published:** 2025-01-02

**Authors:** Hongwu Yu, Yifan Wen, Wenqian Yu, Liang Lu, Yu Yang, Chengye Liu, Zhixiang Hu, Zhuting Fang, Shenglin Huang

**Affiliations:** 1 Department of Integrative Oncology, Fudan University Shanghai Cancer Center, and Shanghai Key Laboratory of Medical Epigenetics, Institutes of Biomedical Sciences, Fudan University, Shanghai, 200032, China.; 2 Department of Oncology, Shanghai Medical College, Fudan University, Shanghai, 200032, China.; 3 Department of Oncology and Vascular Interventional Therapy, Clinical Oncology School of Fujian Medical University, Fujian Key Laboratory of Translational Cancer Medicine, Fujian Cancer Hospital (Fujian Branch of Fudan University Shanghai Cancer Center), Fuzhou, 350014, China.; 4 Department of Interventional Radiology, Shengli Clinical Medical College of Fujian Medical University, Fujian Provincial Hospital, Fuzhou University Afliated Provincial Hospital, Fuzhou 350001, China.

**Keywords:** Circular RNA, Cancer vaccine, Tumor neoantigen, Internal ribosome entry sites (IRES), Human papillomavirus (HPV) cancer vaccine

## Abstract

**Rationale:** Circular RNA (circRNA) has gained attention as a promising platform for mRNA vaccines due to its stability, sustained protein expression, and intrinsic immunostimulatory properties. This study aimed to design and optimize a circRNA cancer vaccine platform by screening for efficient internal ribosome entry sites (IRES) and enhancing circRNA translation efficiency for improved cancer immunotherapy.

**Methods:** We screened 29 IRES elements to identify the most efficient one for immune cell translation, ultimately discovering the *Enterovirus* A (EV-A) IRES. Using SHAPE-MaP technology, we analyzed the secondary structure of circRNA and introduced targeted mutations and deletions to optimize translation efficiency. Additionally, we investigated the regulatory roles of spacer sequences and microRNA recognition sites in circRNA design and examined the mechanisms behind IRES-mediated translation initiation.

**Results:** The EV-A IRES was identified as the most efficient for immune cell translation. Structural modifications and optimization of spacer sequences enhanced the translation efficiency of circRNA. Comparative studies demonstrated that circRNA vaccines induced stronger T cell immune responses and exhibited superior tumor prevention and therapeutic efficacy compared to traditional linear mRNA vaccines.

**Conclusion:** The optimized tumor antigen circRNA vaccine platform offers a stable, efficient alternative to conventional mRNA vaccines for cancer immunotherapy, with enhanced immune responses and improved therapeutic outcomes. This work lays the foundation for developing circRNA-based vaccines as a novel strategy for cancer treatment.

## Introduction

Immunotherapy has emerged as a highly effective strategy in cancer treatment, gaining widespread recognition in the field of oncology 1. Among the various forms of immunotherapy, vaccines have been explored for cancer prevention and treatment for several decades. However, it is only in recent years that mRNA-based cancer vaccines have shown promising clinical outcomes 2,3. This advancement can be attributed, in part, to the rapid deployment of mRNA vaccine technology during the COVID-19 pandemic 4, which significantly accelerated the clinical research and development of mRNA cancer vaccines. mRNA is now considered one of the most promising platforms for cancer vaccines for several key reasons. First, mRNA vaccines have demonstrated an excellent safety profile, as evidenced by the large-scale administration of COVID-19 mRNA vaccines. Second, mRNA has intrinsic immunostimulatory properties, and mRNA vaccines have shown superior immunogenicity compared to DNA, protein, and peptide vaccines. Finally, mRNA vaccines can be rapidly designed and manufactured, making them particularly well-suited for the development of personalized neoantigen cancer vaccines. These attributes position mRNA as a highly advantageous platform for advancing cancer vaccine development 5.

Extensive research on nucleotide modifications, cap analogs, and RNA sequence optimization has substantially enhanced the pharmacological potential of traditional linear mRNA. Currently, 5'-cap 1 mRNAs with 1-methyl-pseudouridine (m1ψ) 6 modification, optimal 5' and 3' untranslated sequences (UTRs) 7,8, and polyadenylate (polyA) length 9,10 have been shown to yield superior translation efficiency and stability. Additional strategies to enhance linear mRNA include refining purification protocols to minimize double-stranded RNA (dsRNA) contamination 11,12, optimizing the secondary structure of the coding sequence 13,14, and incorporating branched polyA tails 15. However, these approaches either yield marginal improvements or substantially increase the complexity of production processes without fundamentally enhancing druggability. Furthermore, although m1ψ modification is essential for achieving optimal translation efficiency and stability, recent study has indicated that m1ψ modification may induce frameshift translation 16, thereby presenting potential adverse effects. From a vaccine platform perspective, m1ψ modification significantly diminishes the intrinsic immunogenicity of mRNA, potentially undermining its capacity to elicit an effective immune response. On the other hand, unmodified linear mRNA exhibits suboptimal expression levels, rendering it a less favorable option for vaccine development. Consequently, the development of tumor vaccines utilizing novel mRNA platforms might address these challenges and offer unforeseen advantages.

Due to its high stability and naturally lacking termini, circular RNA (circRNA) is considered a promising mRNA drug platform 17. In recent years, the permuted intron-exon (PIE)-based RNA circularization method has been optimized, making circRNA synthesis simpler and more efficient 18. Most synthetic circRNA incorporates a viral Internal Ribosome Entry Site (IRES) to initiate protein translation 18-20. Consequently, IRES translation efficiency is a key determinant of circRNA protein expression levels, which determines the efficacy of circRNA therapeutics. While previous studies have shown that IRES screening and adjacent sequence optimization can significantly enhance circRNA translation efficiency 20, there has been limited exploration of highly efficient IRES in specific cell types, such as immune cells. Moreover, viral IRES function through multiple domains that recruit translation initiation factors and IRES trans-acting factor (ITAFs) 21-23, suggesting that systematic optimization of these domains could further enhance IRES function. On the other hand, synthetic circRNA, due to the presence of exogenous sequences and large secondary structures, can be recognized by cellular pattern recognition receptors, thereby triggering immune responses 24-26, which could pose challenges in drug development. However, in the context of cancer immunotherapy, drugs with immunostimulatory properties may more effectively induce tumor-targeting immunity. Therefore, stable and immunostimulatory circRNA is likely to be an ideal tumor vaccine platform. Studies have demonstrated that engineered circRNA vaccines can efficiently induce adaptive immunity, resulting in significant tumor suppression in animal models 27,28. Nonetheless, more research is needed to compare the immune induction capability and tumor treatment efficacy of circRNA vaccines with those of well-established linear mRNA vaccines.

In this study, we aim to design a circRNA platform specifically suited for tumor vaccines by optimizing the key translational element, IRES, and systematically comparing it with conventional linear mRNA vaccines *in vitro* and animal models. First, we identified an IRES from *Enterovirus* genus EV-A virus, which demonstrated highest translation efficiency in immune cell lines. We employed SHAPE-MaP **(**selective 2' hydroxyl acylation analyzed by primer extension and mutational profiling**)** technology to resolve its structure and further optimized and streamlined its sequence while screening for the best-performing UTR elements in the circRNA platform. Additionally, we explored the mechanism of IRES-mediated translation initiation, discovering that IRES-mediated cap-independent translation might compete with cap-dependent translation for initiation factors. Ultimately, the circRNA vaccine exhibited superior antigen-specific immune induction and tumor suppression effects in both *in vitro* and *in vivo* animal models compared to traditional linear mRNA vaccines.

## Results

### Screening for highly efficient IRES

**Figure 1:**
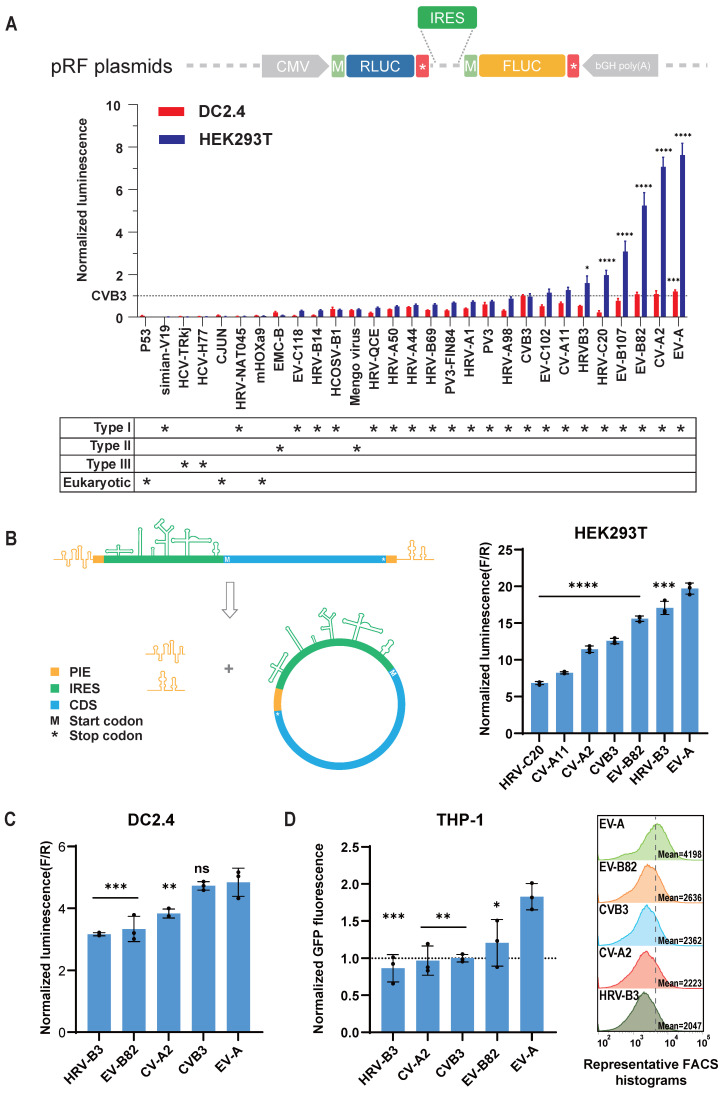
** Screening for highly efficient IRES.** (A) Dual-luciferase reporter assay to assess IRES translation efficiency. Top panel, schematic diagram of the pRF plasmids in which IRESs were inserted between coding sequences of Renilla luciferase (RLUC) and Firefly luciferase (FLUC). Middle panel, translation efficiencies (TEs) in HEK293T and DC2.4 were calculated by RLUC/FLUC luciferase activities, and normalized to the TE of CVB3. Bottom panel, asterisks denote type of IRESs. Dashed line indicates TE of CVB3. The statistical significance was assessed between CVB3 and other IRESs. (B) and (C) Top IRESs were cloned into circular FLUC mRNA and co-transfected with RLUC mRNA into HEK293T (B) and DC2.4 (C) cells. (B) Left panel, schematic diagram of circRNA design and circularization. Right panel, FLUC/RLUC ratio at 24 hours post transfection. (C) FLUC/RLUC ratio at 24 hours post transfection of DC2.4 cells. (D) Top IRESs were cloned into circular EGFP mRNA and screened in THP-1. Left panel, bar plot of GFP signal normalized to CVB3. Right panel, representative FACS histogram of GFP signal. Mean GFP fluorescence of n>100,000 singlet cells per sample was measured by flow cytometry. Dashed line indicates mean GFP signal of EV-A. The statistical significance was assessed between EV-A and other IRESs in (B), (C) and (D). All data are mean (SD) for n= 3 biological replicates. One-way ANOVA, Dunnett's post-test was used to calculate the statistical significance. *P < 0.05 was considered statistically significant. **P < 0.01, ***P < 0.001 and ****P < 0.0001 were considered highly significant. ns, not significant.

CircRNA cannot initiate translation via the 5' cap-dependent mechanism, instead relying on IRES or m6A modifications to initiate translation 29. To enhance the expression levels of circRNA, particularly in immune cells for application as tumor vaccines, we first screened IRES elements with high translation efficiency. Previous studies have shown that viral IRES elements achieve the highest translation efficiency on circRNA 18,20. Thus, our candidate IRES elements were primarily derived from viruses. We screened a total of 29 IRES elements (Figure 1A, Figure S1A, and Table S1), including 22 type I, 2 type II, 2 type III viral IRES, and 3 mammalian cell-derived IRES 30-32. Initially, we utilized dual-luciferase reporter plasmids to compare the cap-independent translation efficiency of these IRES elements in the mouse dendritic cell line DC2.4 and HEK293T cells (Figure 1A). Consistent with previous studies 18,20, type I IRES elements generally exhibited stronger translation efficiency compared to other types and mammalian IRES elements (Figure 1A). As anticipated, the translation efficiency of IRES elements varied between different cell types. For example, the translation efficiency of HRV-C20 was approximately twice that of CVB3 in HEK293T cells, but only 20% of CVB3's efficiency in DC2.4 cells. This screening revealed that the IRES from *Enterovirus* A (EV-A) exhibited the highest translation efficiency in both cell lines.

To further validate the performance of highly efficient IRES elements in circRNA, we incorporated the seven highest-expressing IRES elements into circRNA constructs, synthesizing circular firefly luciferase (FLUC) mRNA using the previously reported PIE circularization method 18 (Figure 1B and Figure S1B). The expression profiles of these IRES elements in circRNA differed from those in the dual-luciferase reporter system, potentially due to unexpected splicing events in plasmid-based system 33, or differences in the spatial configuration of IRES elements in circRNA compared to linear RNA 34. Nonetheless, both in the dual-luciferase plasmid and circRNA contexts, the IRES from EV-A demonstrated the highest relative translation efficiency (Figure 1B). The IRES elements from HRVB3 and CVB3, previously reported for their high translation efficiency 18,20, also performed well but were slightly less efficient than the EV-A IRES in both DC2.4 and HEK293T cells (Figure 1C).

To investigate the adaptability of the EV-A IRES to different coding sequences, we constructed circular EGFP mRNA and compared EGFP expression levels in HEK293T, DC2.4, and the human monocytic cell line THP-1 (Figure 1D, Figure S1C). Our results confirmed that the EV-A IRES maintained the highest translation efficiency. These findings indicate that the EV-A IRES is a robust translation element for circRNA, adaptable to different coding sequences and exhibiting high translation efficiency in mouse and human immune cell lines.

### Structural analysis and sequence optimization of EV-A IRES

Previous studies have elucidated that the five structural domains (domains II to VI) of type I viral IRES recruit translation initiation factors and ITAFs to initiate translation 23. Specifically, domain V directly interacts with eukaryotic initiation factors eIF4G and eIF4A, facilitating the assembly of the 48S ribosomal complex. Domains II and IV recruit hnRNP A1 and PCBP1/2 respectively, stabilizing the IRES structure and aiding in the recruitment of initiation factors. Ribosomal scanning occurs within domain VI, while the function of domain III remains less well understood. We hypothesized that mutating and simplifying the structural domains of the EV-A IRES might stabilize its structure and enhance translation efficiency. Therefore, we employed SHAPE-MaP technology 35 to resolve the structure of the EV-A IRES, designed targeted mutations and truncations, and compared their translation efficiencies.

**Figure 2:**
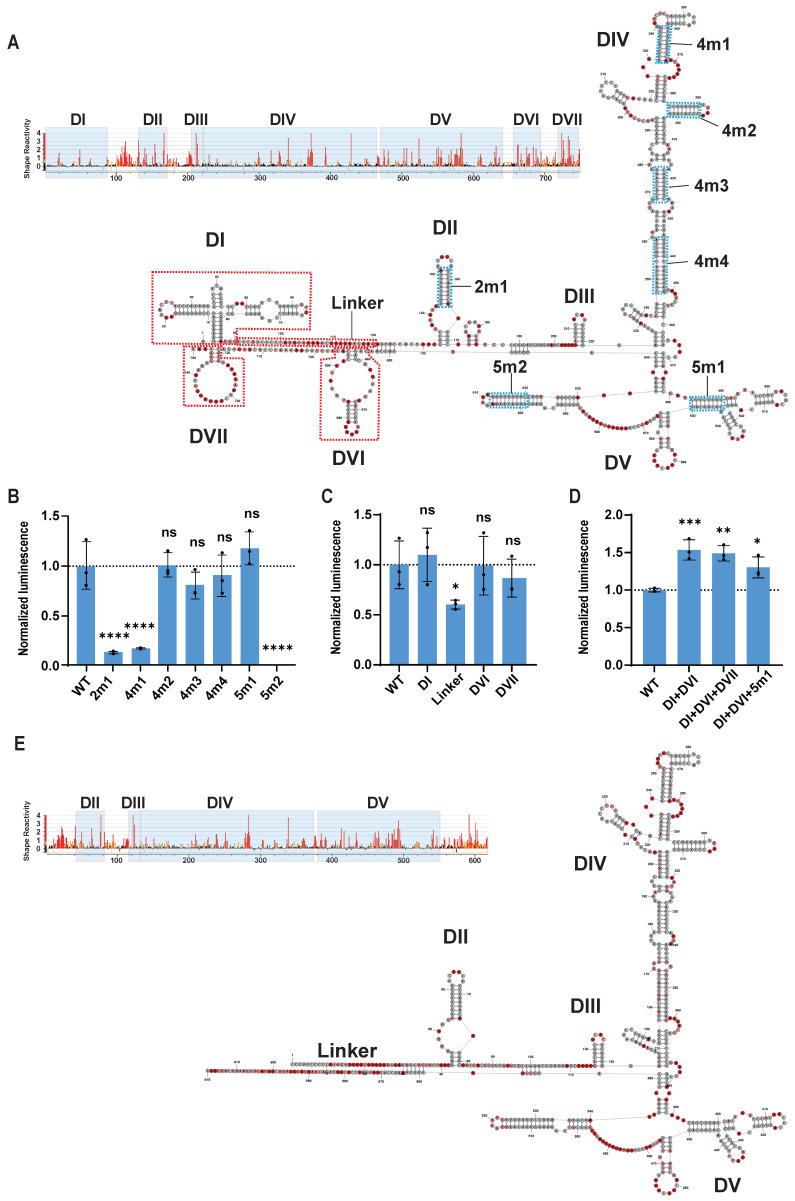
** Secondary structure and mutational modification of EV-A IRES.** (A) SHAPE-MaP reactivity and secondary structure of wild-type EV-A IRES. Upper panel, SHAPE-MaP reactivity profile. Reactivities below 0.4 are colored black, reactivities between 0.4 and 0.85 are shown in orange and reactivities above 0.85 are shown in red. Light blue blocks indicate profile of predicted structures. Lower panel, secondary structure. The nucleotide coloring indicates normalized reactivity values of <=0.4(gray), >0.4 and <0.85 (light red), and >=0.85 (dark red). Bold black characters indicate structural domains. (B) Comparison of translation efficiency between artificially mutated and the wild-type EV-A IRES. (C) Comparison of translation efficiency between truncated mutants and the wild-type EV-A IRES. Mutants were constructed based on circular EV-A GLUC mRNA. Mutants and wild-type mRNAs were transfected into HEK293T, and GLUC activity was measured 24 hours post transfection. GLUC activity was normalized to wild-type EV-A. Mutations (light blue dotted boxes with linked characters) and truncations (red dotted boxes with linked characters) are indicated in (A). (D) Comparison of translation efficiency between combinational mutants (DI+DVI, DI+DVI+DVII and DI+DVI+5m1) and wild-type EV-A IRES. (E) SHAPE-MaP reactivity and secondary structure of DI+DVI mutant EV-A IRES. All data are mean (SD) for n= 3 biological replicates. One-way ANOVA, Dunnett's post-test was used to calculate the statistical significance. *P < 0.05 was considered statistically significant. **P < 0.01, ***P < 0.001 and ****P < 0.0001 were considered highly significant. ns, not significant.

The SHAPE-MaP secondary structure model of the EV-A IRES revealed a typical type I IRES secondary structure, encompassing domains I to VI (Figure 2A). Notably, the EV-A IRES also exhibited a unique stem-loop structure located after domain VI, which we designated as domain VII. Sequence comparisons between EV-A and other *Enterovirus* IRES elements (Figure S2A) indicated that domains I, II, III, IV, and V are highly conserved, whereas domains VI and VII are more variable. Domain I primarily participates in viral genome replication rather than translation. Based on this analysis, our optimization strategy included mutating key functional domains and deleting less conserved or non-essential sequences.

Our approach to optimizing key functional domains was to increase the GC content of long stems to strengthen base-pairing without altering their secondary structure or critical protein-binding regions (e.g., PCBP1/2, eIF4G) 36,37. We designed seven EV-A mutants based on this approach (Figure 2A blue dashed boxes and Table S1), incorporated them into circular gaussia luciferase (GLUC) mRNA, and transfected HEK293T cells to measure GLUC luminescence, thereby comparing the translation efficiencies of the EV-A mutants (Figure 2B). Unfortunately, none of the mutants significantly increased translation efficiency compared to the wild-type EV-A. The 4m2, 4m3 and 4m4 mutations in domain IV and the 5m1 mutation in domain V exhibited no notable impact, whereas the 2m1 mutation in domain II, 4m1 mutation in domain IV, and 5m2 mutation in domain V significantly reduced translation efficiency. These findings suggest that the nucleotide composition of these regions, in addition to secondary structure, plays a crucial role in IRES functionality.

Next, we systematically truncated the longer, non-essential sequences of EV-A, including domain I (90nt), the linker between domains I and II (30nt), domain VI (42 nt), and domain VII (32nt) (Figure 2A red dashed boxes and Table S1). Luminescence signal results indicated that truncating domains I, VI, and VII did not affect IRES translation, whereas deleting the linker significantly reduced translation efficiency (Figure 2C). To assess whether further combinations of truncations might impact IRES function, we generated EV-A mutants with domain I + VI truncations (DI+DVI), domain I+VI+VII truncations (DI+DVI+DVII) and domain I + VI truncations combined with the 5m1 mutation (DI+DVI+5m1). Interestingly, simultaneous truncation of domains I and VI significantly improved EV-A translation efficiency by approximately 50%, while additional truncation of domain VII or incorporation of the 5m1 mutation did not provide further enhancement (Figure 2D). Moreover, the translation efficiency of these three mutants is significantly higher than that of the wild-type EV-A in both DC2.4 and THP-1 cells, confirming that this improvement is cell type-independent (Figure S2B and S2C). We hypothesized that the improvement resulted from the more streamlined and stable structure with both domains I and VI truncated. To verify this hypothesis, we resolved the structure of the DI +DVI mutant. SHAPE-MaP reactivity signals within the core regions of the DI +DVI mutant were almost identical to those of the wild-type EV-A (Figure 2E). Comparing the predicted secondary structures of the DI +DVI mutant and wild-type EV-A IRES, we observed that the structures of the functional domains (domains II, III, IV, V) were perfectly preserved in the mutant. Unexpectedly, not only were the deleted structures (domains I and VI) absent, but a small stem-loop near domain II and domain VII also disappeared (Figure 2E). This result supports our hypothesis that deleting multiple non-essential structures rendered the IRES more streamlined and stable. In summary, our engineering based on SHAPE-MaP structural analysis generated a shorter (reduced from 750 nt to 618 nt) and more stable EV-A IRES with significantly enhanced translation efficiency.

### Screening for spacers and regulatory elements

**Figure 3:**
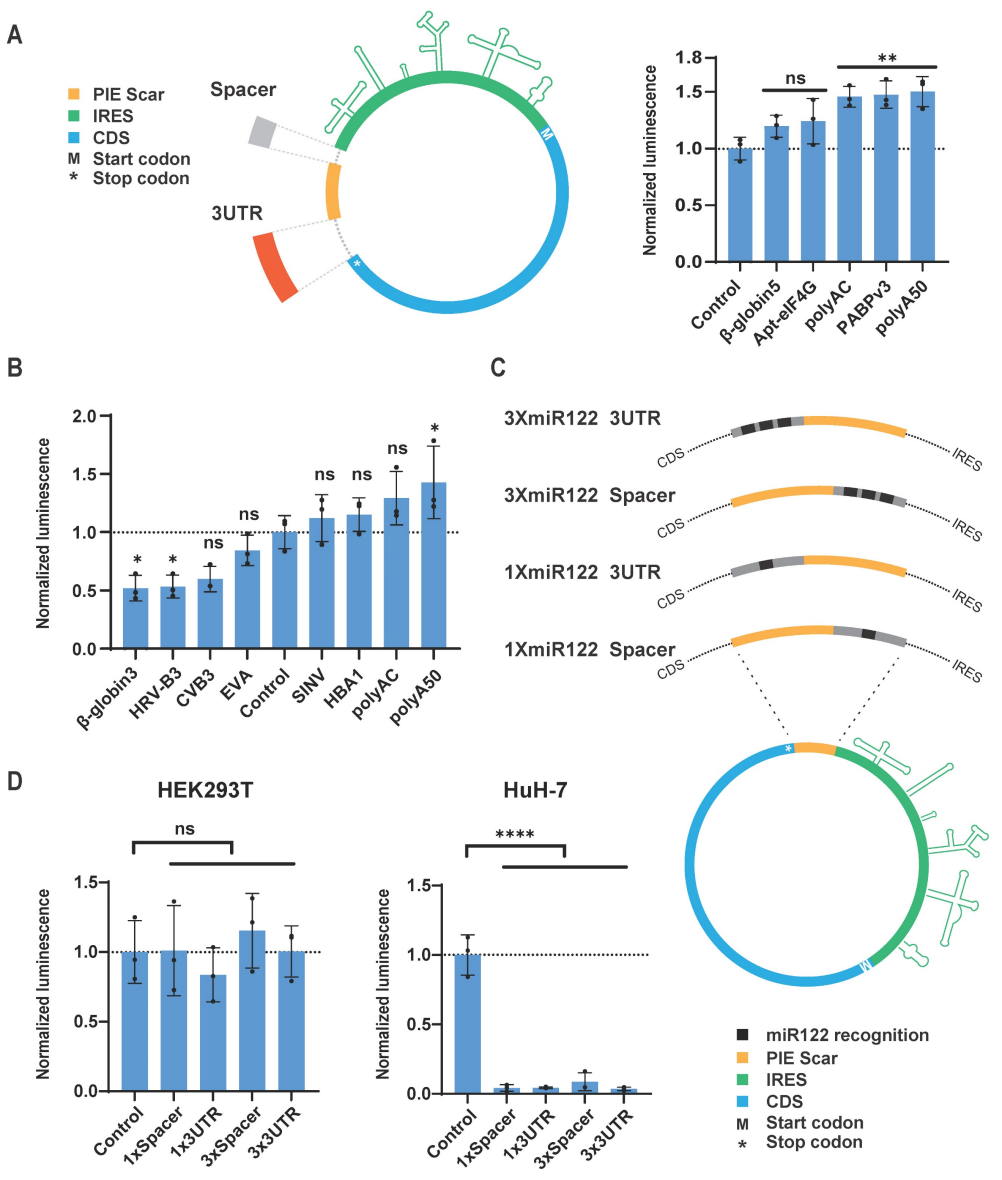
** CircRNA untranslated region screening and introduction of miR122 recognition sequence.** (A and B) Translation efficiency comparison of circular GLUC mRNA containing indicated spacer (A) and 3UTR (B) in HEK293T cells. Left panel of (A), schematic diagram of insertion site of spacer/3UTR in circRNA. (C) Schematic diagram of insertion sites of miR-122 recognition sequence in circular GLUC mRNA. (D) Translation efficiency of circular GLUC mRNA with miR-122 recognition sequence in HEK293T (Left panel) and HuH-7 (Left panel) cells. CircRNAs were transfected into HEK293T or HuH-7 cells, and GLUC activity was measured 24 hours post transfection. GLUC activity was normalized to control circRNA without Spacer or 3'UTR. All data are mean (SD) for n= 3 biological replicates. One-way ANOVA, Dunnett's post-test was used to calculate the statistical significance. *P < 0.05 was considered statistically significant. **P < 0.01, ***P < 0.001 and ****P < 0.0001 were considered highly significant. ns, not significant.

Although translation of circRNA is mediated by IRES, the translation efficiency and expression level of the target protein are also significantly influenced by other sequences within the circRNA design 18,20. For instance, residual sequences from the PIE circularization method may inhibit circRNA translation. However, incorporating spacer sequences between the IRES, CDS, and residual sequences can mitigate such inhibition 20. Similar to the 5' UTR and 3' UTR of classical linear mRNA, spacer sequences can differentially impact the translation and stability of circRNA depending on their relative positions to the IRES and CDS. Therefore, we refer to the spacer sequences at the 5' end of the IRES and the 3' end of the CDS as the 5' Spacer and 3' UTR of the circRNA, respectively, and we screened these sequences separately (Figure 3A and Table S2).

We also utilized circular GLUC mRNA (circGLUC) to screen for optimal UTR sequences. For the spacer sequences, we compared PABP v3, apt-eIF4G 20, beta-globin 5' UTR 7, polyAC 18, and 50nt of polyadenylate (polyA50) to the control circGLUC without spacer or 3' UTR. The results indicated that polyA50 yielded the highest GLUC signal when used as the 5' Spacer (Figure 3A). For the 3' UTR, our screening included hemoglobin subunit alpha 1 (HBA1) 3' UTR, polyAC, polyA50, and beta-globin 3' UTR 7. Additionally, considering that viral 3' UTR sequences may facilitate IRES function 38, we also included the 3' UTR sequences from CVB3, EV-A, HRV-B3, and SINV in our screening. The results showed that polyA50 was also the most effective spacer sequence in the 3' UTR position (Figure 3B). Overall, the polyA50 sequence promoted IRES function most effectively, whether used as the 5' Spacer or the 3' UTR, likely due to its simple spatial structure and its ability to recruit PABP, promoting the interaction between IRES and the translation initiation factor eIF4G.

In the design of circRNA, in addition to adding spacer sequences to promote protein expression, incorporating microRNA recognition sequences to regulate circRNA expression in specific tissues is also a meaningful strategy. miR-122 is a liver-specific microRNA highly expressed in the liver 39. Studies have demonstrated that incorporating miR-122 recognition sites into AAV vectors successfully inhibited AAV vector expression in the liver 40. We added sequences containing miR-122 recognition sites to the 5' Spacer or 3' UTR positions of circRNA and transfected them into the miR-122-expressing liver cancer cell line HuH-7 (Figure 3C and Table S2). Both 1× and 3× miR-122 recognition sites in either position significantly reduced GLUC expression in HuH-7, whereas in HEK293T cells, which do not express miR-122, GLUC expression showed no significant difference compared to control circRNA without spacers (Figure 3D). This result indicates that adding just one recognition site sequence to circRNA can achieve substantial miR-122-mediated knockdown of circRNA. These findings suggest that microRNA recognition sites can be added to circRNA to enable effective tissue-specific regulation of circRNA distribution.

### Regulation of CircRNA translation and *in vitro* immune induction of CircRNA vaccine

Previous studies have indicated that the translation initiation mechanism of viral IRES differs from that of eukaryotic mRNA 5' cap. For instance, the 5' cap recruits eIF4E, followed by eIF4G, forming the eIF4F complex, which subsequently recruits the 43S pre-initiation complex 21,41. In contrast, type I IRES recruits eIF4G with the assistance of ITAFs, bypassing the requirement for eIF4E 42. Similarly, the translation initiation mechanism of *in vitro* transcribed circRNA with IRES is expected to differ significantly from that of *in vitro* transcribed linear mRNA with 5' cap analogs. To compare the translation initiation mechanisms of these two mRNA platforms, we designed siRNAs targeting key translation regulatory proteins, including PABP1, ITAFs PCBP1 and PCBP2, and translation initiation factors eIF4E, eIF4G1, eIF4G2, and eIF3D (Table S4).

**Figure 4:**
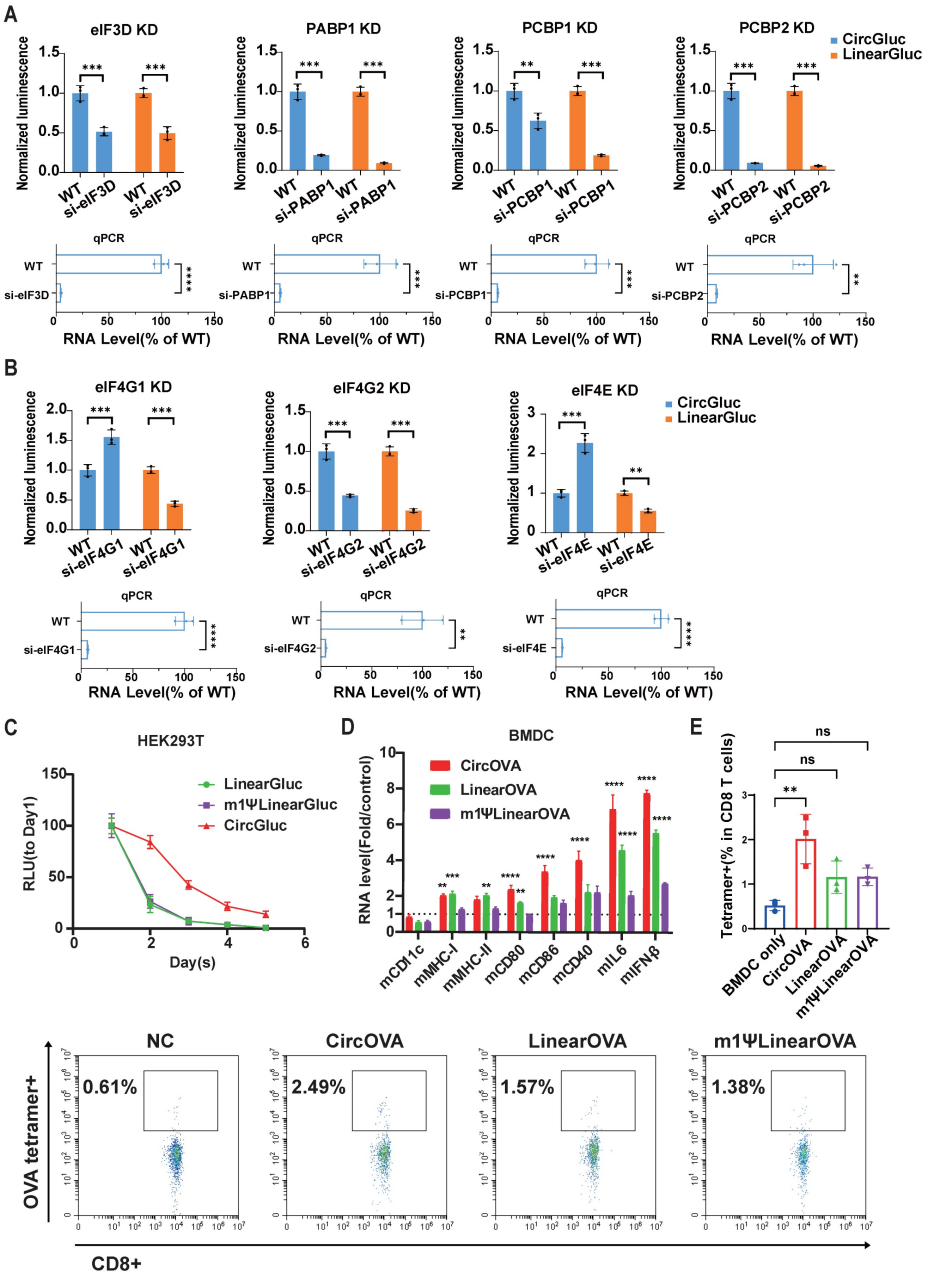
** CircRNA translation regulation and *in vitro* OVA-specific T cell expansion.** (A and B) Translation efficiency of circular and linear GLUC mRNA after knockdown of PABP1/PCBP1/PCBP2 (A), eIF4G1/ eIF4G2/ eIF4E/ eIF3D (B). HEK293T cells were treated with siRNA targeting the indicated genes for 48 hours. Treated cells were then transfected with circular or linear mRNA, and GLUC activity was measured 24 hours post transfection. Upper panel, GLUC activity normalized to wild-type control experiment treated with negative control siRNA. Lower panel, normalized RNA level of corresponding genes measured by qPCR. (C) GLUC activity of the HEK293T culture supernatant though day 1 to day 5 after transfection of circular, linear and m1Ψ modified linear GLUC mRNA. (D) Normalized RNA level of corresponding genes in BMDC cells after transfection of circular, linear or m1Ψ modified linear OVA mRNA. RNA level was normalized to mock transfected control group. (E) OVA-specific T cell expansion after co-culturing with BMDCs transfected with circular, linear or m1Ψ modified linear OVA mRNA. Upper panel, quantified percentages of OVA-specific T cells. Lower panel, representative flow cytometry diagram. All data are mean (SD) for n= 3 biological replicates. In (A) and (B), Unpaired two-sided t-test was used to calculate the statistical significance. In (D), two-way ANOVA was used. In (E), One-way ANOVA was used. *P < 0.05 was considered statistically significant. **P < 0.01, ***P < 0.001 and ****P < 0.0001 were considered highly significant. ns, not significant.

Upon knockdown of PABP1, PCBP1, PCBP2, eIF3D, and eIF4G2, both circRNA and linear RNA translation were significantly reduced (Figure 4A, 4B). Interestingly, knockdown of cap-dependent translation initiation factors eIF4E and eIF4G1 resulted in a significant decrease in linear RNA translation but a notable increase in circRNA translation (Figure 4B). These results suggest that circRNA translation shares common regulatory proteins with cap-dependent translation, but circRNA may also compete with cellular cap-dependent translation for common initiation factors. This might require additional attention in future studies on the safety and efficacy of circRNA therapeutics. From another perspective, hijacking initiation factors specific for cap-dependent translation may further elevate circRNA translation.

We further compared the stability and immune induction capabilities of circRNA and linear mRNA to evaluate whether circRNA offers advantages in these areas. Consistent with previous studies 18,20, our circRNA exhibited prolonged and enhanced expression compared to both modified and unmodified linear mRNA (Figure 4C and Figure S3A). Additionally, some studies have reported that circRNA possesses strong immunostimulatory properties, potentially acting as an adjuvant to enhance adaptive immunity 24-27. To assess the immune induction capability of circRNA, we transfected BMDC cells with circular, unmodified linear, and modified linear mRNA encoding the Ovalbumin (OVA) antigen (Table S3). Eight hours post-transfection, we measured the expression of BMDC activation-related genes via RT-qPCR. The results showed that BMDCs transfected with circular OVA expressed the highest levels of DC activation-related genes (CD80, CD86, CD40), IL6 and type I interferon signal (IFN β), followed by those transfected with unmodified linear OVA, indicating that circRNA has a stronger capacity to activate DCs (Figure 4D).

Subsequently, we co-cultured BMDCs transfected with these three types of OVA mRNA with mouse spleen cells containing a small proportion of OVA-specific T cells (0.5% of all CD8+ T cells). Six days later, we measured the proportion of OVA-specific T cells and found that the circRNA group induced greatest proliferation of OVA-specific T cells (Figure 4E). These results demonstrate that circRNA can induce specific T cell immunity more robustly, suggesting that circRNA-based tumor vaccines may have superior efficacy compared to traditional linear mRNA vaccines.

### Tumor prophylactic effects of circular RNA vaccines

**Figure 5:**
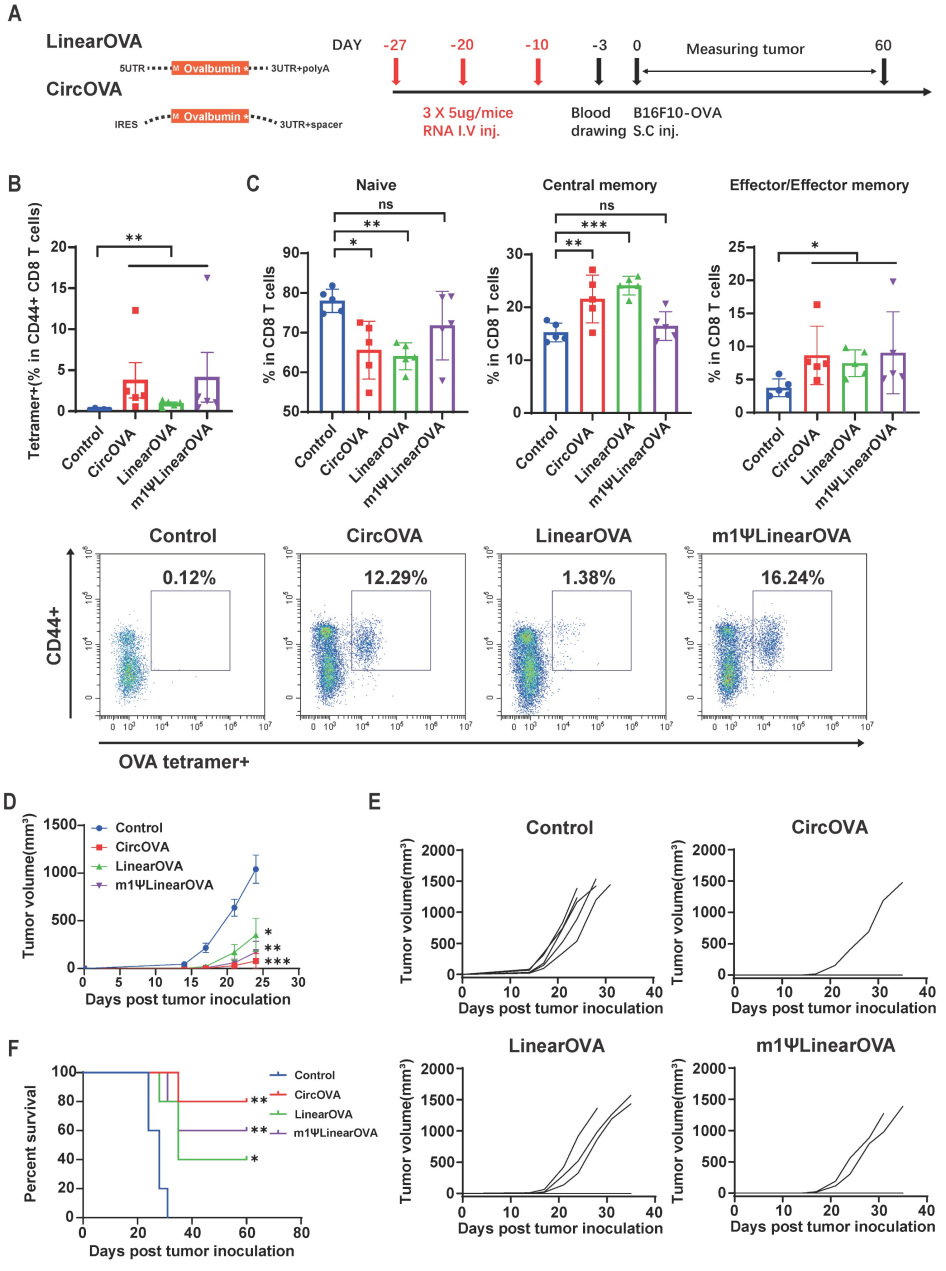
** Circular OVA mRNA vaccine induced effective T cell response and protective effect against B16F10-OVA tumor model.** (A) Timeline of vaccination, drawing blood and monitoring of tumor volume. Mice were injected retro-orbitally with CircOVA(n=5)/LinearOVA(n=5)/ m1ΨLinearOVA(n=5) vaccine three times (5μg per dose) in 17 days. 1x10^5^ B16F10-OVA cells were injected subcutaneously to each mouse 10 days after the third vaccination. (B) Representative flow cytometry diagram and percentages of OVA-specific T cells in PBMC 7 days after the third vaccination. (C) Quantified percentages of naïve (CD44-CD62L+), central-memory (CD44+CD62L+) and effector/effector-memory (CD44+CD62L-) T cells within CD8+ T cells in PBMC 7 days after the third vaccination. (D) Tumor volumes util day 24 after tumor inoculation. (E) Tumor volume of individual mice until day 35. (F) Survival rate. For (D), data are mean (SEM). All other data are mean (SD). In (B) and right panel of (C), Mann-Whitney U test was used to calculate the statistical significance. In (D) and left and middle panel of (C), one-way ANOVA was used. In (F) Kaplan-Meier simple survival analysis was used to calculate the survival rate, and Log-rank (Mantel-Cox) test was used to calculate the statistical significance. *P < 0.05 was considered statistically significant. **P < 0.01, ***P < 0.001 and ****P < 0.0001 were considered highly significant. ns, not significant.

To evaluate the tumor prophylactic effects of our designed circRNA platform as cancer vaccine, we selected OVA as a model antigen and established a B16F10 stable cell line expressing OVA through lentivirus infection. Mice were vaccinated with three doses of the circular or linear OVA mRNA vaccines via intravenous injection on days -27, -17, and -10 before subcutaneous inoculation with B16F10-OVA tumor cells. Blood samples were collected three days before tumor inoculation to analyze vaccine induced systematic T cell response in PBMCs (Figure 5A). Flow cytometry analysis showed that the proportion of OVA peptide (SIINFEKL) MHC tetramer-positive T cells in PBMCs was significantly higher in the three vaccinated groups compared to the untreated group, indicating that all three types of RNA vaccines successfully induced OVA-specific T cells (Figure 5B). Notably, the CircOVA and m1ψLinearOVA vaccines induced a higher proportion of OVA-specific T cells compared to the LinearOVA vaccine.

Further analysis of T cell subsets in PBMCs revealed that the proportions of naïve T cells were significantly lower in the CircOVA and LinearOVA groups compared to the untreated group, whereas the m1ψLinearOVA group showed no significant difference from the untreated group (Figure 5C). The proportion of central memory T cells was significantly higher in the CircOVA and LinearOVA groups, while all three vaccine groups exhibited a significantly higher proportion of effector T cells compared to the untreated group (Figure 5C). These results suggest that CircOVA and unmodified LinearOVA vaccines shifted overall T cell composition towards a more activated phenotype, possibly due to recognition by pattern recognition receptors in immune cells enhanced overall antigen presentation. In contrast, the m1ψLinearOVA vaccine, with its lower intrinsic immunostimulatory effect, imposed smaller alterations on the overall T cell subset composition.

Ten days after the third vaccination, mice were subcutaneously inoculated with B16F10-OVA tumor cells, and tumor growth was monitored for 60 days post-inoculation. In the vaccinated groups, some mice did not develop tumors through the 60-day observation period (CircOVA: 4/5, m1ψLinearOVA: 3/5, LinearOVA: 2/5). Notably, 80% of the mice in the CircOVA group remained tumor-free, whereas all mice in the untreated group developed tumors within approximately two weeks (Figure 5E, 5F). On day 24 post-tumor inoculation, the tumor sizes in the vaccinated groups were significantly smaller than those in the untreated group, with some tumors in the untreated group reaching the humane endpoint (Figure 5D). These results demonstrate that our circRNA vaccine exhibits excellent immune activation capability, comparable antigen specific T cell induction ability to modified linear mRNA, and superior tumor prophylactic effect.

### Therapeutic efficacy of circular RNA vaccines targeting tumor neoantigens and tumor-associated antigens

**Figure 6:**
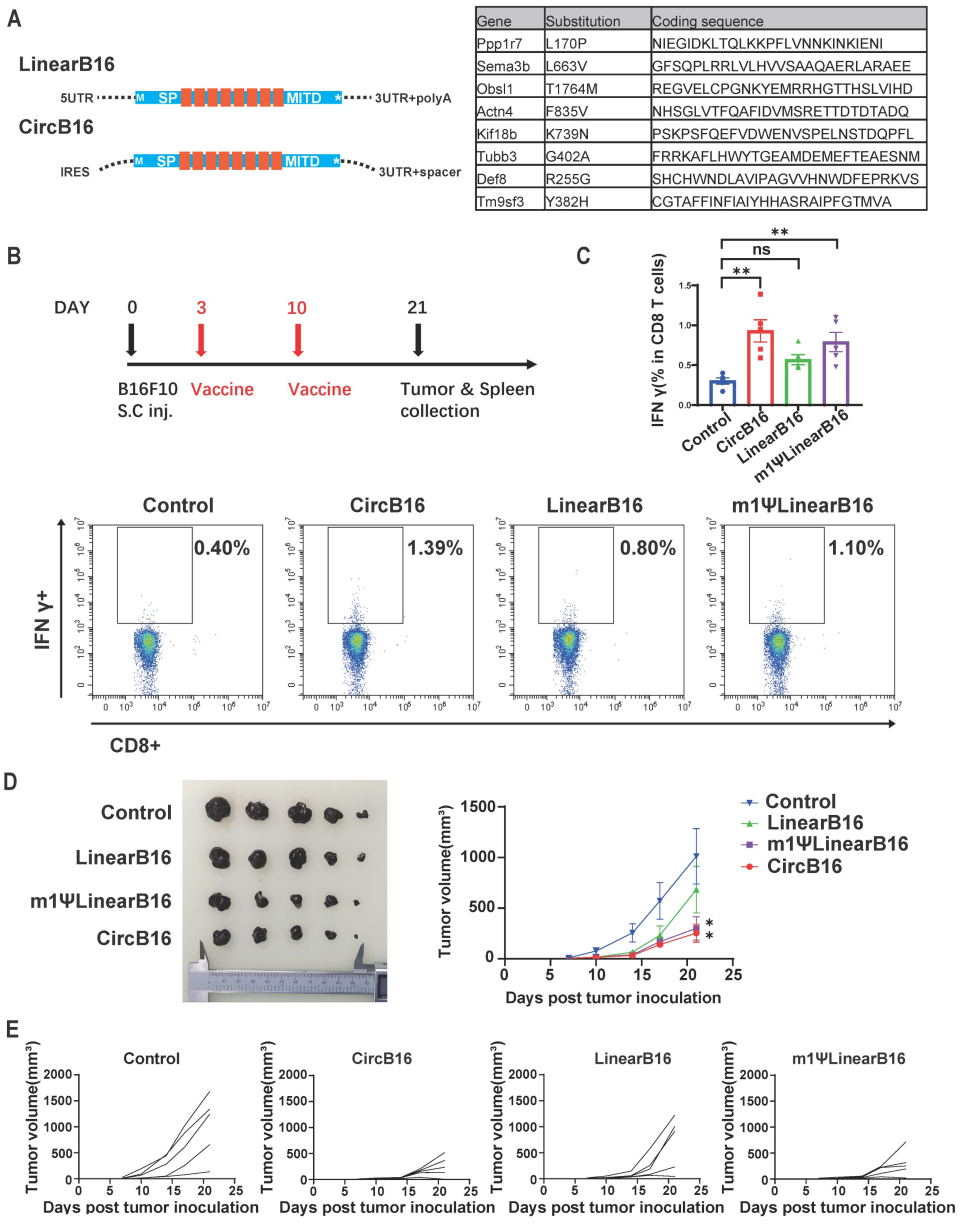
** CircRNA encoded neoantigen vaccine induced strong neoantigen specific T cell response and optimal therapeutic effect against B16F10 tumor.** (A) Design of circular and linear B16F10 neoantigen vaccines. Left panel, schematic diagram of the concatemer vaccine design. Signal peptide (SP) and transmembrane domain (MITD) of human MHC-I were added to augment antigen presentation. Right panel, target information of the 8 previously reported B16F10 neoantigen encoded by the vaccines. (B) Timeline of vaccination, monitoring of tumor volume and collecting tumor and spleen samples. Mice were subcutaneously inoculated with 1x10^5^ B16F10 cells and vaccinated retro-orbitally with CircB16-8(n=5)/LinearB16-8 (n=5)/m1ΨLinearB16-8 (n=5) on day 3 and 10 (5μg per dose). (C) Representative flow cytometry diagram and percentages of B16-8 antigen-specific T cells in splenocyte. Splenocytes were stimulated with BMDCs transfected with m1ΨLinearB16-8 mRNA for 16 hours, then stained for intra-cellular IFNγ. (D) Tumors collected on day 21 and tumor volumes until day 21 after tumor inoculation. (E) Tumor volume of individual mice until day 21. For (D), data are mean (SEM). All other data are mean (SD). In (C) and (D), one-way ANOVA was used to calculate the statistical significance. *P < 0.05 was considered statistically significant. **P < 0.01, ***P < 0.001 and ****P < 0.0001 were considered highly significant.

**Figure 7:**
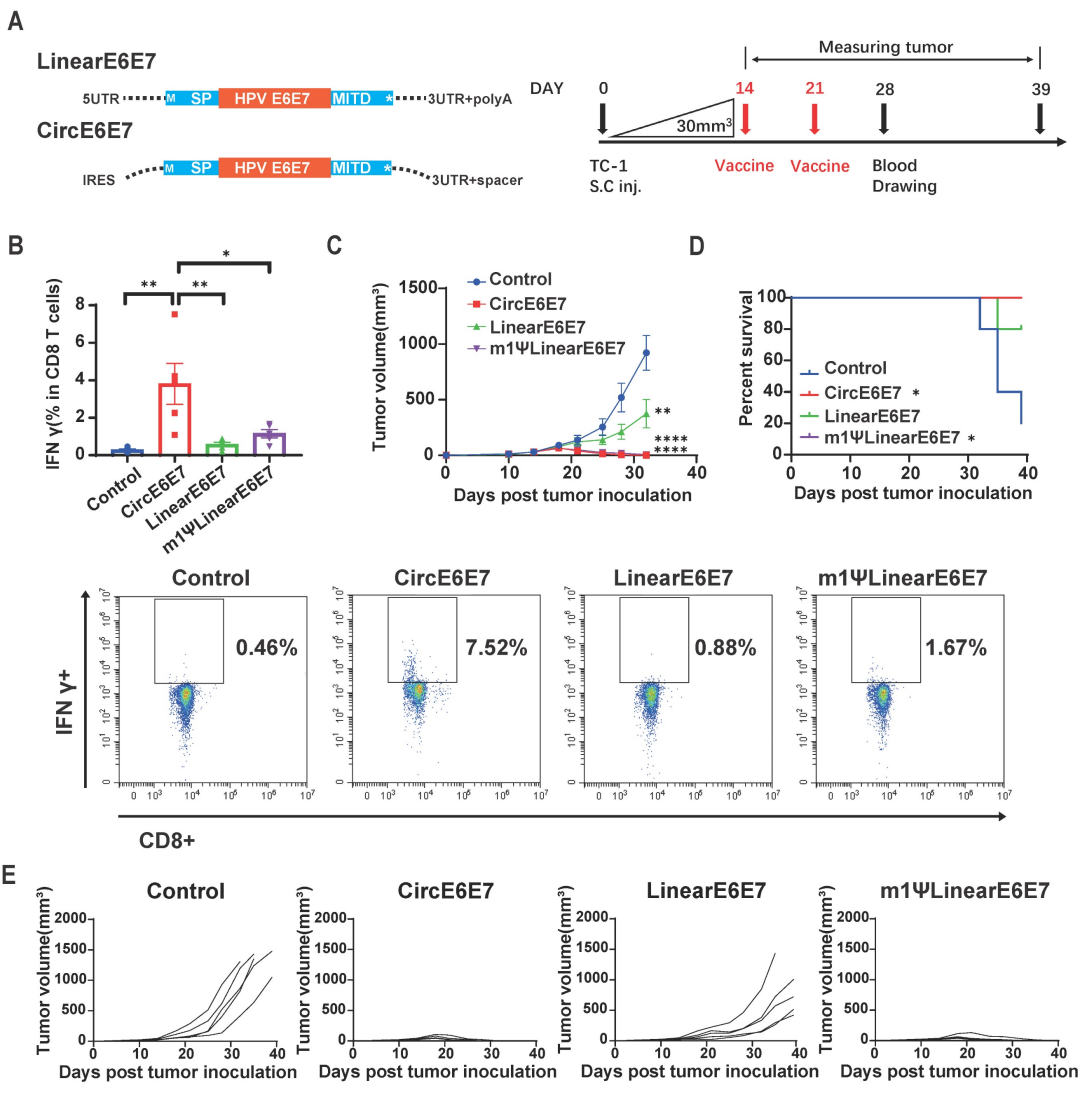
** Circular HPV antigen vaccine induced eradication of HPV associated TC-1 tumor model.** (A) Design of circular and linear HPV E6+E7 fusion protein vaccines. Left panel, schematic diagram of the vaccine design. A fusion protein of the HPV E6, E7 oncogenes reported by previous study was used as the encoding antigen. Signal peptide (SP) and transmembrane domain (MITD) of human MHC-I were added to augment antigen presentation. Right panel, timeline of vaccination, monitoring of tumor volume and drawing blood. Mice were subcutaneously inoculated with 1x105 TC-1 cells. When average tumor volume reached about 30mm^3^ on day 14, mice were vaccinated retro-orbitally with CircE6E7 (n=5)/Linear E6E7 (n=5)/m1ΨLinear E6E7 (n=5, 5μg per dose), and received a booster dose on day 21 (5μg per dose). (B) Representative flow cytometry diagram and percentages of E6E7 antigen-specific T cells in PBMC. PBMCs were stimulated with mixed MHC class I restricted E6 and E7 epitopes (2.5 μg/ml each; EVYDFAFRDL for E6, RAHYNIVTF for E) for 16 hours, and stained for intra-cellular IFNγ. (C) Tumor volumes until day 32 after tumor inoculation. (D) Survival rate. (E) Tumor volume of individual mice until day 39. For (C), data are mean (SEM). All other data are mean (SD). In (B) and (C), one-way ANOVA was used to calculate the statistical significance. In (D) Kaplan-Meier simple survival analysis was used to calculate the survival rate, and Log-rank (Mantel-Cox) test was used to calculate the statistical significance. *P < 0.05 was considered statistically significant. **P < 0.01, ***P < 0.001 and ****P < 0.0001 were considered highly significant.

OVA is a highly immunogenic xenogeneic animal antigen, and therefore, the B16F10-OVA model may not accurately reflect the clinical scenario of tumor treatment. Tumor mutational neoantigens and virus-derived tumor-associated antigens are common targets in tumor vaccine development. To evaluate the therapeutic potential of the circRNA vaccines, we designed vaccines encoding a tandem of eight reported B16F10 tumor mutational neoantigens 44,45 (Figure 6A and Table S3) and an HPV E6E7 fusion protein vaccine 46 (Figure 7A and Table S3), and tested them in B16F10 and TC-1 tumor models, respectively.

Three days after subcutaneous inoculation of B16F10 cells, mice were immunized with the three types of B16-8 tandem antigen vaccines (Figure 6B). A booster immunization was administered on day 10, and tumor growth were monitored until day 21. On day 21, mice were euthanized, and spleens and tumors were collected. To assess the proportion of vaccine-induced neoantigen-specific T cells, we measured the proportion of IFN-γ+ CD8+ T cells in splenocytes stimulated with BMDCs transfected with m1ψLinearB16 mRNA. The results showed that all three vaccine groups induced higher levels of IFN-γ+ CD8+T cells, with the CircB16-8 group showing the highest average proportion (Figure 6C). Tumor monitoring results also indicated that both CircB16 and m1ψLinearB16 vaccines significantly inhibited tumor growth (Figure 6D, 6E).

The TC-1 cell line, which stably expresses HPV oncogenes E6 and E7, is commonly used as an HPV-related tumor model. To evaluate the therapeutic efficacy of the HPV tumor vaccine on established tumors, we inoculated mice subcutaneously with TC-1 cells and, after 14 days (average tumor volume reached 30 mm^3^), administered mRNA vaccines encoding the HPV E6/E7 fusion protein via intravenous injection. Blood samples were collected seven days after the second vaccine dose (Figure 7A). Flow cytometry analysis of PBMCs revealed that the CircE6E7 vaccine induced significantly greater proportion of IFN-γ-secreting T cells in PBMCs stimulated with E6 and E7 derived peptides compared to all other groups (Figure 7B). Both the m1ψLinear E6E7 and Linear E6E7vaccine induced higher proportion of antigen specific T cells compared to the control group, although these differences were not statistically significant. Consistent with the antigen-specific T cell induction results, the CircE6E7 and m1ψLinearE6E7 vaccines successfully eradicated the tumors, whereas the LinearE6E7 vaccine also inhibited tumor growth but to a lesser extent (Figure 7C, 7D and 7E). Taken together, these results demonstrate that the circRNA vaccines encoding tumor mutational neoantigens and HPV antigens induced stronger tumor antigen-specific T cell immunity and exhibited superior therapeutic efficacy in mice compared to conventional linear mRNA vaccines.

## Discussion

To design and optimize a tumor circRNA vaccine platform, this study specifically screened for highly efficient IRESs in immune cell lines, and identified the EV-A IRES. After resolving its secondary structure, we engineered a streamlined mutant of EV-A IRES with significantly higher translation efficiency through a series of mutation and deletion assessments. We also screened for more suitable untranslated sequences to pair with our IRES and tested the introduction of microRNA recognition sites for precise regulation of circRNA. Our circRNA-encoded vaccines induced higher levels of T cell immune responses compared to conventional linear mRNAs in both *in vitro* immune cell models and *in vivo* animal models, demonstrating strong tumor prevention and therapeutic effects.

The IRES is the primary translation initiation element in circRNA and determines the expression levels of circRNA therapeutics, making its screening and optimization a crucial step in circRNA drug development. In 2018, Wesselhoeft *et al.* compared several viral and cellular IRES elements on circRNA and identified the CVB3 IRES as the most efficient 18. A more extensive screening and modification was conducted by Chen *et al.* in 2023 20, who employed a modular construction platform to compare the efficiencies of dozens of IRES elements, ultimately identifying HRV-B3 as more efficient. However, previous studies did not specifically screen for highly efficient IRES elements in immune cells for use in tumor circRNA vaccines. In our study, screening 29 IRESs elements revealed that EV-A exhibited the highest translation efficiency in immune cell lines DC2.4 and THP-1, as well as in HEK293T cells.

We further analyzed the structure of EV-A using SHAPE-MaP technology and made targeted mutations and deletions of each domain, successfully simplifying and enhancing the translation efficiency of EV-A. Structural analysis of the simplified EV-A revealed that the key functional structures was perfectly retained, while all non-core structures, not limited to the deleted ones, disappeared. This potentially indicates that the simplified IRES became more stable, thereby improving translation efficiency. Additionally, we discovered that although the linker sequence before domain II did not form a secondary structure, its deletion significantly reduced translation efficiency, suggesting a functional role in translation. Interestingly, mutations in different sequences within the functional domains, without affecting the secondary structure, had varying impacts on translation, indicating distinct function of these sequences. For instance, some sequences within the functional domains may responsible for secondary structure formation while others interact with proteins or distal sequences through their specific nucleotides. Overall, our results show that IRES needs to retain only the core domains to function effectively in translation initiation, with the secondary structure and nucleotide composition of the core domains both playing crucial roles. However, our analysis of the structure and function of EV-A IRES is still limited, especially concerning the engineering and investigation of core functional domains. Future research with more comprehensive and high-throughput engineering of EV-A IRES core domains could provide a deeper understanding of the relationship between its structure and function, and further elevate its efficiency.

Moreover, we compared the translation regulation mechanisms of linear mRNA and circRNA. We found that PABP1, PCBP1, PCBP2, eIF3D, and eIF4G2 support the translation of both linear and circular mRNA. However, knockdown of cap-dependent translation initiation factors eIF4E and eIF4G1 resulted in increased circRNA translation but decreased linear mRNA translation, indicating their primary involvement in cap-dependent translation. Although eIF4G2, a member of the eIF4G family, lacks the eIF4E-binding domain present in eIF4G1 47, it is generally considered to participate in cap-independent translation mechanisms such as IRES, CITE, and m6A 48. However, it has also been found to affect cap-dependent translation efficiency 49, which explains why its reduction impaired translation of both RNA types. The opposite effects on translation efficiency for the two types of RNA following eIF4E and eIF4G1 knockdown suggest competition between IRES-mediated and cap-dependent translation for downstream initiation factors. This implies that exogenous circRNA entering cells may compete with endogenous mRNA for translation initiation proteins. Similarly, exogenous linear mRNA also inevitably competes with endogenous mRNA for translation-related proteins. This could be a safety concern for both mRNA therapeutics. Nonetheless, reasonably utilizing the differences and competitive relationships in these translation mechanisms might be an effective way to further enhance circRNA translation levels.

Despite our results showing that circRNA vaccines have stronger immunostimulatory capabilities compared to linear mRNA vaccines, we did not delve into the underlying mechanisms. Notably, circRNA has a tendency to spontaneously break, making it difficult to rule out the possibility that the resulting 5'-monophosphate linear RNA fragments could trigger immune responses. In our circRNA production process, RNaseR treatment is used to remove free intron and full-length pre-RNA byproducts from the mixture, but a small amount of nicked linear RNA fragments may still remain (Figure S1B and Figure S7). Due to the spontaneous breaking of circRNA, other purification methods like High Performance Liquid Chromatography (HPLC) or gel extraction used in other studies also leave some nicked RNA fragments 18,26,50. Previous studies have varied views on whether circRNA itself activates cellular immune responses: Wesselhoeft *et al.* suggested that circRNA does not activate immune responses due to the absence of ends 51; Chen *et al.* proposed that exogenous sequences in circRNA activate immune responses, which can be suppressed by m6A modification 24,25; Liu *et al.* demonstrated that stem-loop structures of different lengths in circRNA have distinct effects on immune responses 26. A recent study indicated that both circRNA itself and impurities in its production process are recognized by different pattern recognition receptors, resulting in immune activation 50. Cells recognize exogenous RNA in various ways, including end recognition and double-stranded RNA recognition. The complex RNA double-stranded structures of the IRES in circRNA could potentially be recognized by cells, triggering immune responses 24-27. Activation of cellular immune responses could be a disadvantage in certain application contexts. However, when dose, efficacy and safety are properly balanced, this property of circRNA could be a significant advantage in application such as cancer immunotherapy.

Several studies have recently explored the use of circRNA to encode tumor-targeting drugs. For example, circRNA has been used to encode cytokines such as IL-15 and IL-12 for the treatment of melanoma and liver cancer, either as standalone therapies or in combination with PD-1 antibodies 51,52. Another significant area of research is the use of circRNA as a tumor vaccine platform. Hongjian Li *et al.* were the first to develop a circRNA-based tumor vaccine, creating a lipid nanoparticle system for circRNA delivery. Their circRNA vaccine effectively induced both adaptive and innate immunity and significantly inhibited tumor growth 28. In 2023, Laura Amaya *et al.* showed that circRNA inherently possesses adjuvant activity, effectively enhancing T-cell immune responses. Their circRNA vaccine encapsulated by charge-altering releasable transporters (CARTs) also exhibited strong anti-tumor efficacy 27. These findings are consistent with our data in this study, confirming that circRNA can indeed enhance the immune response. Most recently, Fei Wang *et al.* designed a circRNA-based liver cancer vaccine, which exhibited more stable expression compared to linear mRNA and demonstrated superior efficacy in treating solid tumors 53. Collectively, these studies, along with our findings, consistently support the idea that circRNA, with its stable expression and adjuvant activity, is an ideal platform for tumor vaccines.

As we highlighted above, circRNA has emerged as a promising RNA drug platform in recent years, yet it still requires significant advancements in production, safety, and efficacy research. Despite these challenges, its unique properties and promising preclinical results justify more comprehensive and in-depth investigations. Future studies should aim to elucidate the mechanisms underlying circRNA translation regulation and immune responses, as well as to advance the design and clinical applications of circRNA. It is anticipated that the advantages of circRNA drugs will soon be realized in the treatment of tumors and various other diseases.

## Materials and Methods

### Molecular cloning

For dual luciferase reporter assay, IRESs are chemically synthesized and cloned into a pRF plasmid between the RLUC and FLUC coding sequences. The pUC57 plasmids, inserted with the T7 promoter, Anabaena 2.0 PIE elements reported by Wesselhoeft *et al.* 18, IRESs and coding sequences through seamless cloning were used as the circRNA template plasmids. The Cloning Kit for mRNA Template (Takara Bio) was used to construct all the linear mRNA templates. The template vector in that kit contains a T7 promoter, a human beta-globin 5' UTR, a human HBA1 3' UTR and a 105nt long poly(A) sequence. DNA fragments were synthesized by Genewitz (Suzhou, China) and amplified by PCR. ClonExpress II One Step Cloning Kit form Vazyme (Nanjing, China) were used for seamless cloning. The IRES sequences are provided in Table S1.

### Luciferase reporter assay

For dual luciferase reporter assay**,** 100ng of pRF plasmids containing different IRESs were transfected into 10,000 HEK293T or DC2.4 cells/100μL per well of a 96-well plate using Hieff Trans Liposomal Transfection Reagent (Yeasen). 24 hours later, cells were lysed and luminescence counts were detected using Dual Luciferase Reporter Gene Assay Kit (Yeasen) according to manufacturer's instructions. Ratio of FLUC/RLUC counts was considered as IRES activity.

For firefly luciferase assay of circular FLUC RNA, cells were cultured in 96 well plate at the density of 5,000 cells per well. The next day, circular FLUC RNA were transfected with TransIT-mRNA Transfection Kit (Mirusbio) at 50ng per well according to manufacturer's instruction. 24 hours later, cells were lysed on ice for 5 min, both firefly and renilla luminescence counts were measured using Dual Luciferase Reporter Gene Assay Kit (Yeasen) according to manufacturer's instruction. The translation efficiency was calculated by FLUC/RLUC ratio.

For gaussia luciferase assay, cells were cultured in 96 well plate at the density of 5,000 cells per well. The next day, mRNAs were transfected with TransIT-mRNA Transfection Kit (Mirusbio) at 50ng per well according to manufacturer's instruction. 24 hours later, 20μL cell culture medium was taken from each well, and luminescence counts were measured using Gaussia Luciferase Reporter Gene Assay Kit (Beyotime) according to manufacturer's instruction.

### circRNA and linear mRNA synthesis

For the circRNAs, PCR amplified templates were used in in-vitro transcription assay using T7 High Yield RNA Synthesis Kit (Yeasen). The reaction mixture was incubated overnight (~16 hours) at 37°C for maxim spontaneous circularization, and treated with DNase I at 37°C for 20 mins. Then 1×T4 RNA Ligase Reaction Buffer and 2mM (final concentration) GTP was added, and the reaction mixture was incubated at 55°C for 15min to further circularize. RNA was purified with GeneJET RNA Purification Kit, then heated at 65°C for 3min. For every 20ug purified RNA, 20U RNase R (Beyotime) and 1×RNase R reaction buffer was added to the reaction and incubated at 37°C for 2 hours. CircRNA was finally purified with GeneJET RNA Purification Kit, and analyzed by 4% PAGE gel or 2% agarose gel.

For linear mRNAs used in this research, HindIII enzyme linearized plasmids were used as the templated in in-vitro transcription using T7 High Yield RNA Synthesis Kit for Co-transcription (Yeasen). The mRNAs were capped with Cap1 co-transcriptionally and transcribed with a 105nt long polyA tail. After 2 hours incubation at 37°C, the reaction crudes were treated with DNase I at 37°C for 20 mins, then purified with GeneJET RNA Purification Kit (Thermo Scientific) and analyzed by 2% agarose gel.

### SHAPE-MaP (selective 2' hydroxyl acylation analyzed by primer extension and mutational profiling)

Circular EVA-GLUC mRNA was analyzed by SHAPE-MaP method reported by Smola *et al.* 35. Briefly, RNA was modified in 100mM 1-methyl-7-nitroisatoic anhydride (1M7) for 5min under 37°C, then purified with GeneJET RNA Purification Kit (Thermo Scientific). Unmodified and denatured control were prepared the same as Smola's protocol. Purified RNA was fragmented, then reverse transcribed with Hieff NGS Ultima Dual-mode mRNA Library Prep Kit (Yeasen) with some modifications including incubating under 42°C for 3 hours and adding 15 mM MnCl2 (final concentration). cDNAs were then purified with GeneJET RNA Purification Kit (Thermo Scientific). Subsequent RNA library construction was carried out according to the manufacturer's instruction. The sequencing data in FASTQ format was subsequently analyzed by ShapeMapper program according to Smola's protocol. Secondary structure prediction constrained by SHAPE reactivity were generated using RNAstructure program.

### siRNA knockdown

HEK293T cells were cultured in 24 well plates at 100,000 cells per well. siRNAs targeting corresponding proteins were transfected at 30pmol per well with Hieff Trans siRNA/miRNA reagent (Yeasen). 48 hours later, total RNAs of three of the replicate wells were extracted and assayed by RT-qPCR to analysis knockdown efficiency. mRNAs were transfected into the other three replicates, at 200ng per well with TransIT-mRNA Transfection Kit (Mirusbio). 24 hours later, 30μL cell culture medium was taken from each well to measure gaussia luciferase signal. siRNA sequences were synthesized by Hipobio (Huzhou, Zhejiang) and provided in Table S4.

### RNA extraction and RT-qPCR

Total RNA was extracted using EZ-press RNA Purification Kit (EZBioscience) following the manufacturer's instructions. cDNA was synthesized from 500ng total RNA using Evo M-MLV Reverse Transcription Kit (Accurate Biotechnology, Huan) following the manufacturer's instructions. mRNA levels were quantified using SYBR Green Premix Pro Taq HS qPCR Kit (Accurate Biotechnology, Huan), normalized to *Actin*. Primer sequences are provided in Table S5. All qPCR reactions were performed on QuantStudio 5 Real-Time PCR System (Applied Biosystems).

### Western blotting

Circular, modified linear and unmodified linear antigen mRNAs (OVA, B16 and E6E7) were transfected into HEK293T cells in 24-well plate at 400ng/well with TransIT-mRNA Transfection Kit (Mirusbio). 24 hours later, cells are lysed by SDS-loading buffer. Total proteins were separated on a 10% sodium dodecyl sulfate-polyacrylamide gel and transferred on to a nitrocellulose membrane (Bio-Rad, Hercules, CA, USA). The membrane was next blocked with 5% non-fat milk and incubated with Anti- DYKDDDDK (flag) or Anti-actin antibodies (Proteintech). The protein bands were detected using a Tanon 5200 Chemiluminescent Imaging System (Tanon, Shanghai, China) with Omni-ECL reagents (Epizyme Biotech, China).

### BMDC and T cell *in vitro* stimulation

BMDCs were generated with the method reported by Lutz *et al.* 54 For BMDC stimulation assay, BMDCs were cultured in 24 well plates at 250,000 cells per well with mGM-CSF (Peprotech) added to 4ng/mL. 6 hours later, linear or circular mRNAs were transfected at 500ng per well using CALNP mRNA *in vitro* reagent (D-nano, Beijing). 8 hours later, total RNAs were extracted and assayed by RT-qPCR.

For T cell *in vitro* stimulation, BMDCs were cultured in 48 well plates at 200,000 cells per well with mGM-CSF (Peprotech) added to 4ng/mL. 6 hours later, linear or circular OVA mRNAs were transfected at 500ng per well with CALNP mRNA *in vitro* reagent (D-nano, Beijing). 12 hours later, 1 million splenocytes with 0.5% OVA specific CD8 T cell were added to each well, and co-cultured with BMDC for 3 days. On day 3, floating cells were transferred to a new plate, with mIL-7 (Peprotech) and mIL-15 (Peprotech) added to final concentration 5 ng/mL. After another 3 days' culture, cells were analyzed by flow cytometry.

### Animal model

Male C57BL/6N mice aged between 6-8 weeks were purchased from Beijing Vital River Laboratory Animal Technology. For immunization, the mice were injected retro-orbitally with 100μL mRNA (5ug per dose) formulated with *in vivo*-jetRNA reagent (Polyplus). B16F10-OVA cells were generated by lentiviral infection with lentivirus containing pLVX-Puro-OVAL-c plasmid purchased from Shanghai Kambio. For the B16F10-OVA model, 1x10^5^ B16F10-OVA cells in 200ul RPMI 1640 medium (without fetal bovine serum) were subcutaneously injected into the right flank of C57BL/6N mice. For the B16F10 model, 1x10^5^ B16F10 cells in 200ul RPMI 1640 medium (without fetal bovine serum) were subcutaneously injected into the right flank of C57BL/6N mice. For the HPV associated TC-1 model, 1x10^5^ TC-1 cells in 200ul RPMI 1640 medium (without fetal bovine serum) were subcutaneously injected into the right flank of C57BL/6N mice. Mice body weight and tumors were measured twice every week. Tumor volume (V) was determined by using the formula V = L×W^2^/2. All experiments were performed in accordance with protocols approved by Fudan University Experimental Animal Care Commission.

### Flow cytometry analysis

For *in vitro* OVA T cell stimulation assay, the cells were stained with Fixable Viability Dye eFluor 450 (65-0863-14, eBioscience), anti-CD3(APC-65077, Proteintech), anti-CD8a (FITC-65069, Proteintech), OVA Tetramer-SIINFEKL (PE, Helixgen Guangzhou). For OVA mRNA vaccinated mice, peripheral blood was collected through submandibular sampling and lymphocytes were separated by Mouse 1× Lymphocyte Separation Medium (DAKEWE, Shenzhen) according to the manufacturer's instruction. Cells were then stained with Fixable Viability Dye eFluor 450 (65-0863-14, eBioscience), anti-CD3 (APC-65077, Proteintech), anti-CD8a (FITC-65069, Proteintech), anti-CD44 (Proteintech), anti-CD62L (Proteintech), OVA Tetramer-SIINFEKL (PE, Helixgen Guangzhou). For B16F10 neoantigen mRNA vaccinated mice, splenocytes were co-cultured with BMDCs transfected with m1ψLinearB16 mRNA for 16 hours, with brefeldin A (5 μg/ml, Yeasen) and monensin (Yeasen) at the beginning. Then the splenocytes were stained for intra-cellular IFNγ. For HPV E6E7 mRNA vaccinated mice, cells were cultured with E6 and E7 derived peptides (5ug/ml each, GenScript), brefeldin A (5 μg/ml, Yeasen) and monensin (Yeasen) for 16 hours, then stained for intra-cellular IFNγ. For intra-cellular IFNγ staining, cells were first stained with Fixable Viability Dye eFluor 450 (65-0863-14, eBioscience), anti-CD3 (APC-65077, Proteintech), anti-CD8a at 4°C for 30min. Subsequently, cells were fixed and permeabilized with Intracellular Fixation and Permeabilization Buffer Set (eBioscience) according to the manufacturer's instruction, and stained with anti-IFNγ (PE, Biolegend) at room temperature for 20min.

### Capillary electrophoresis analysisof antigen CircRNA

CircRNA treated or untreated by RNaseR were analyzed by Qsep100 (BIOptic) using R1 Cartridge (BIOptic) according to the manufacturer's instruction.

### Statistical analysis

All data in this study were analyzed via Graphpad Prism and presented as mean (SD) unless otherwise indicated. Two-tailed unpaired Student's t-test was used to compare two groups. One-way and two-way ANOVA tests were used to compare more than two groups. Mann-Whitney U test was applied if data sets failed the Pearson omnibus normality test (alpha = 0.05). For survival curves, the data was performed via Kaplan-Meier analysis. *P < 0.05 was considered statistically significant. **P < 0.01, ***P < 0.001 and ****P < 0.0001 were considered highly significant. ns, not significant.

## Supplementary Material

Supplementary figures.

Supplementary tables.

## Abbreviations

mRNA: messenger RNA
IRES: internal ribosome entry sites
circRNA: circular RNA
SHAPE-MaP: selective 2' hydroxyl acylation analyzed by primer extension and mutational profiling
m1ψ: 1-methyl-pseudouridine
UTR: untranslated sequences
polyA: polyadenylate
