# Supplementary material for: Optimized circular RNA vaccines for superior cancer immunotherapy
Source: Theranostics. 2025 Jan 2;15(4):1420–38. doi: 10.7150/thno.104698 (PMC11729565; doi:10.7150/thno.104698)
Supplement: Supplementary file 1 — Supplementary figures. [file thnov15p1420s1.pdf]

Figure S1

A

IRES type and origin

Sequence Source	IRES Type	Virus Genus	Virus species	Virus/Genes
NCBI Virus	Type I	*Enterovirus*	*EnterovirusA (EV-A)*	CV-A2
				simianV19
				EV-A
			EnterovirusC (EV-C)	CV-A11
				EV-C118
				EV-C102
			RhinovirusA (HRV-A)	HRV-A44
				HRV-A98
				HRV-A50
			RhinovirusC (HRV-C)	HRV-NAT045
				HRV-QCE
				HRV-C20
	Type II	*Cardiovirus*	*Encephalomyocarditis virus(EMCV)*	EMC-B
				Mengo virus
	Type III	*Hepacivirus*	*HepacivirusC (HCV)*	HCV-H77
				HCV-Tr Kj
Previous studies	Type I			HRV-B69
				HRV-B14
				HCOSV-B1
				HRV-A1
				EV-B82
				PV3-FIN84
				EV-B107
				HRV-B3
				PV3
				CVB3
	Eukaryotic IRES			P53
				CJUN
				mHOXa9

B

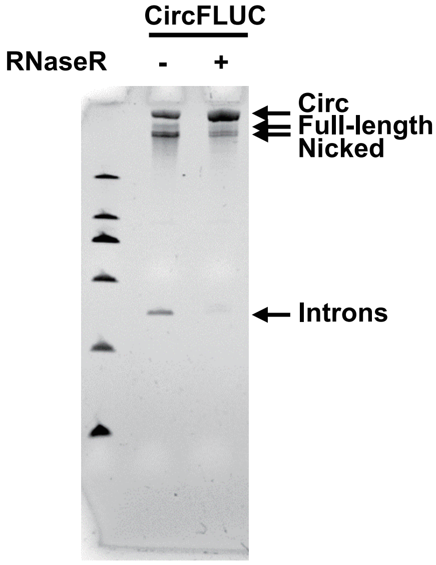

C

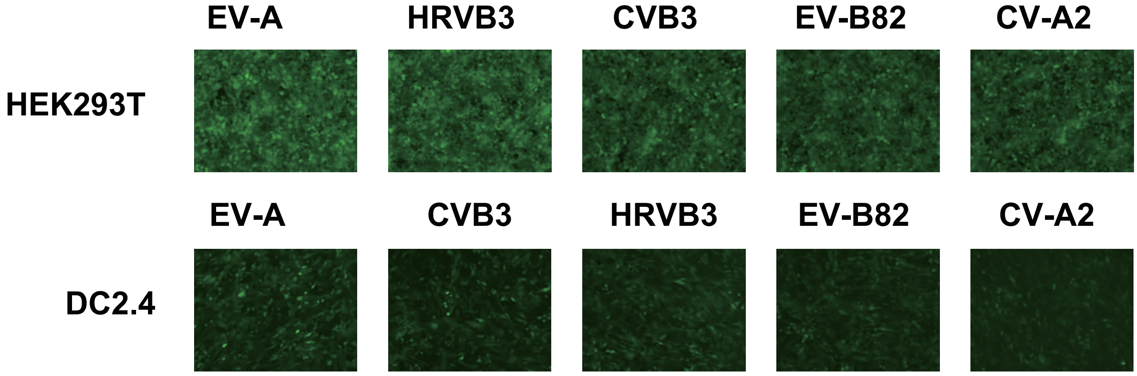

Figure S1. IRES information, gel image of circRNA and IRES comparison by EGFP circRNA. (A) Detailed type and origin of IRESs screened in Figure 1A (B) 4% PAGE gel analysis of CircFLUC circRNA. Lanes left to right are DL2000 DNA marker, untreated CircFLUC and RNase R treated CircFLUC. (C) GFP fluorescence photos of HEK293T and DC2.4 cells transfected with respective IRES. Photos were taken by Olympus fluorescence microscope at an exposure time of 80ms.

Figure S2

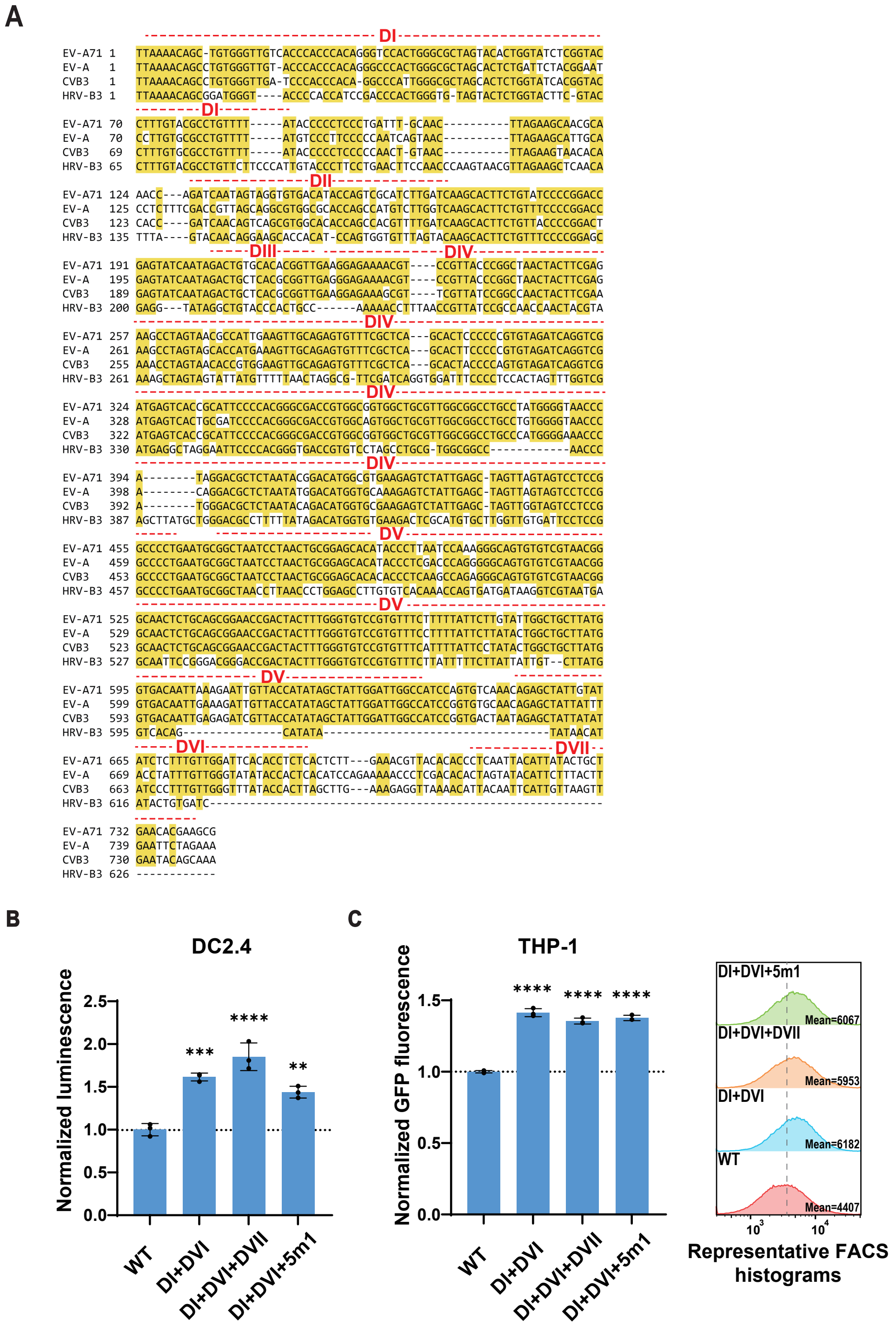

Figure S2. IRES Sequence alignment of four *Enterovirus* genus viruses and translation efficiency of EV-A mutants in DC2.4 and THP-1 cells.

(A) Sequence alignment of EV-A71, EV-A, CVB3 and HRV-B3 IRESs. Dashed line and characters in red indicated regions of the EV-A IRES domains. (B) Comparison of translation efficiency between combinational mutants (DI+DVI, DI+DVI+DVII and DI+DVI+5m1) and wild-type EV-A IRES in DC2.4 cells. (C) Comparison of translation efficiency between circEGFP with EV-A combinational mutants and circEGFP with wild-type EV-A IRES in THP-1 cells. Data are mean (SD) for n= 3 biological replicates. One-way ANOVA, Dunnett's post-test was used to calculate the statistical significance. *P < 0.05 was considered statistically significant. **P < 0.01, ***P < 0.001 and ****P < 0.0001 were considered highly significant. ns, not significant.

Figure S3

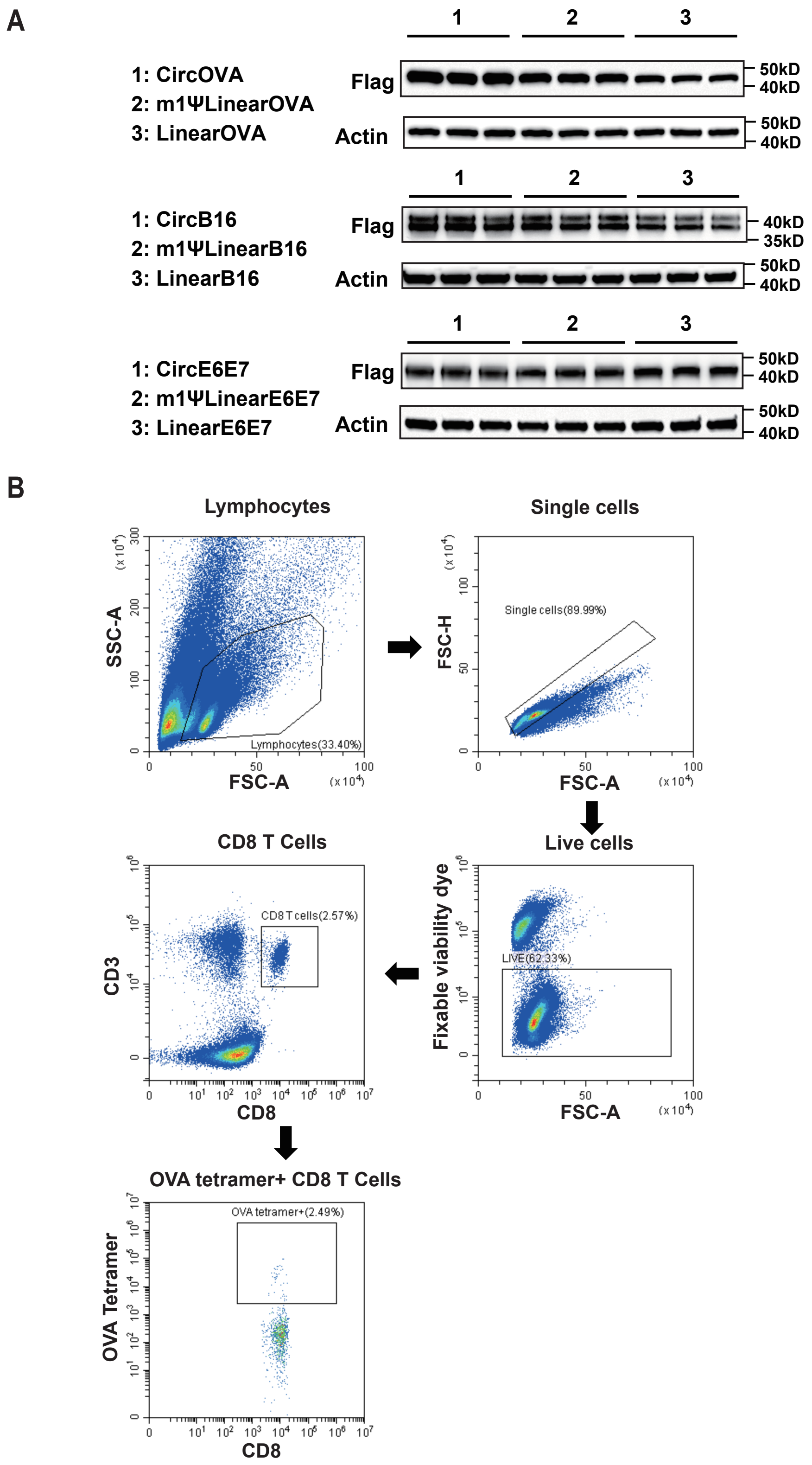

Figure S3. Antigen RNA expression assessed by western blot and Gating strategy of Figure 4. (A) Western blot results of flag-tagged OVA, B16 and E6E7 antigen proteins. Circular, modified linear and unmodified linear antigen mRNAs were transfected into HEK293T cells in 24-well plate at 400ng/well. Total proteins were assessed by western blot 24 hours post transfection. (B) Gating strategy of the flow cytometry data for detecting the percentage of OVA specific CD8+ T cells in Figure 4E.

Figure S4

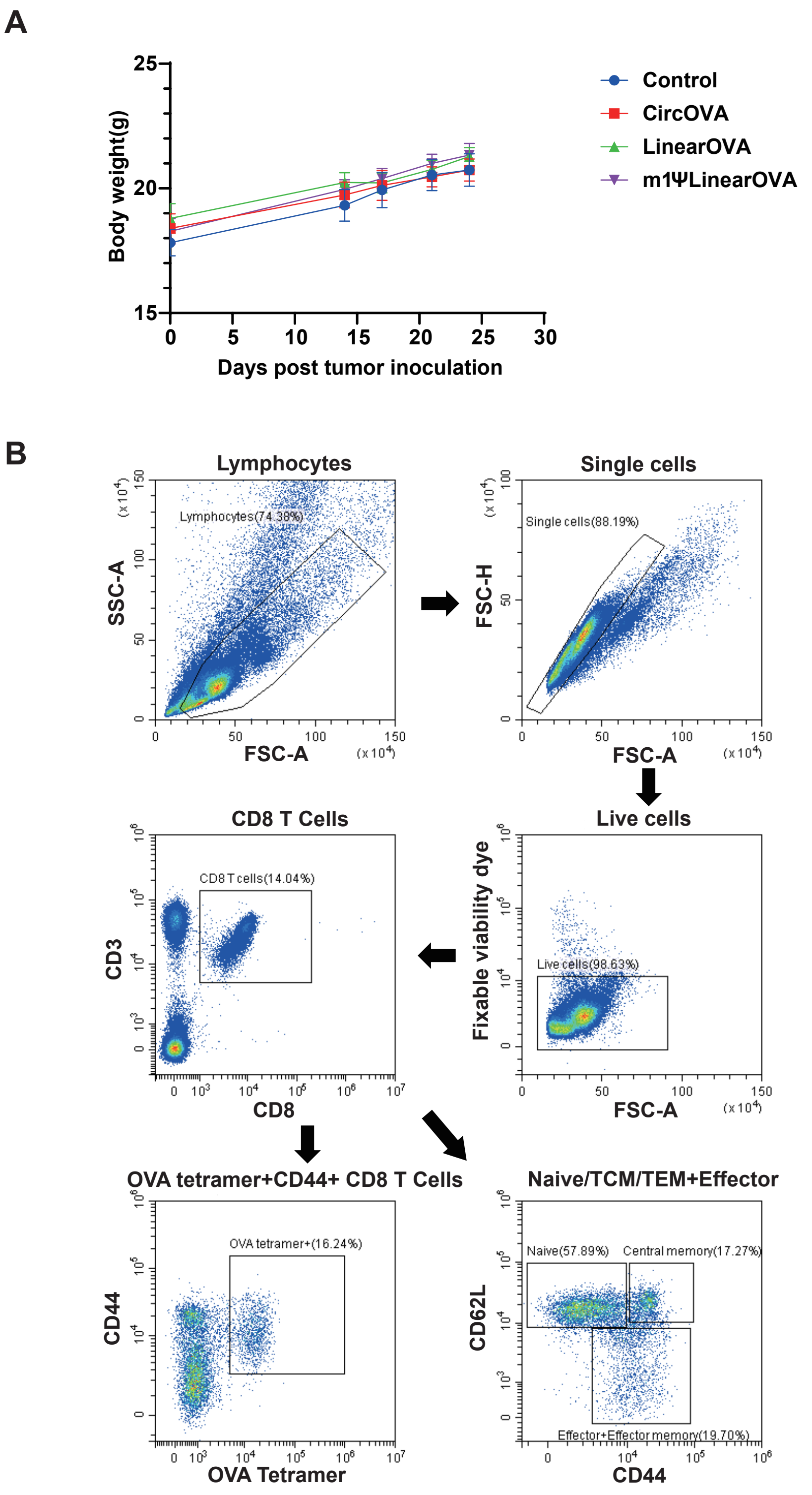

Figure S4. Body weight and gating strategy of Figure 5.

(A) Average body weight of the mice in B16F10-OVA model. (B) Gating strategy of the flow cytometry data for detecting the percentage of OVA specific CD8+ T cells in Figure 5B, and the percentage of naïve (CD44-CD62L+), central-memory (CD44+CD62L+) and effector/effector-memory (CD44+CD62L-) CD8 T cells in Figure 5C.

Figure S5

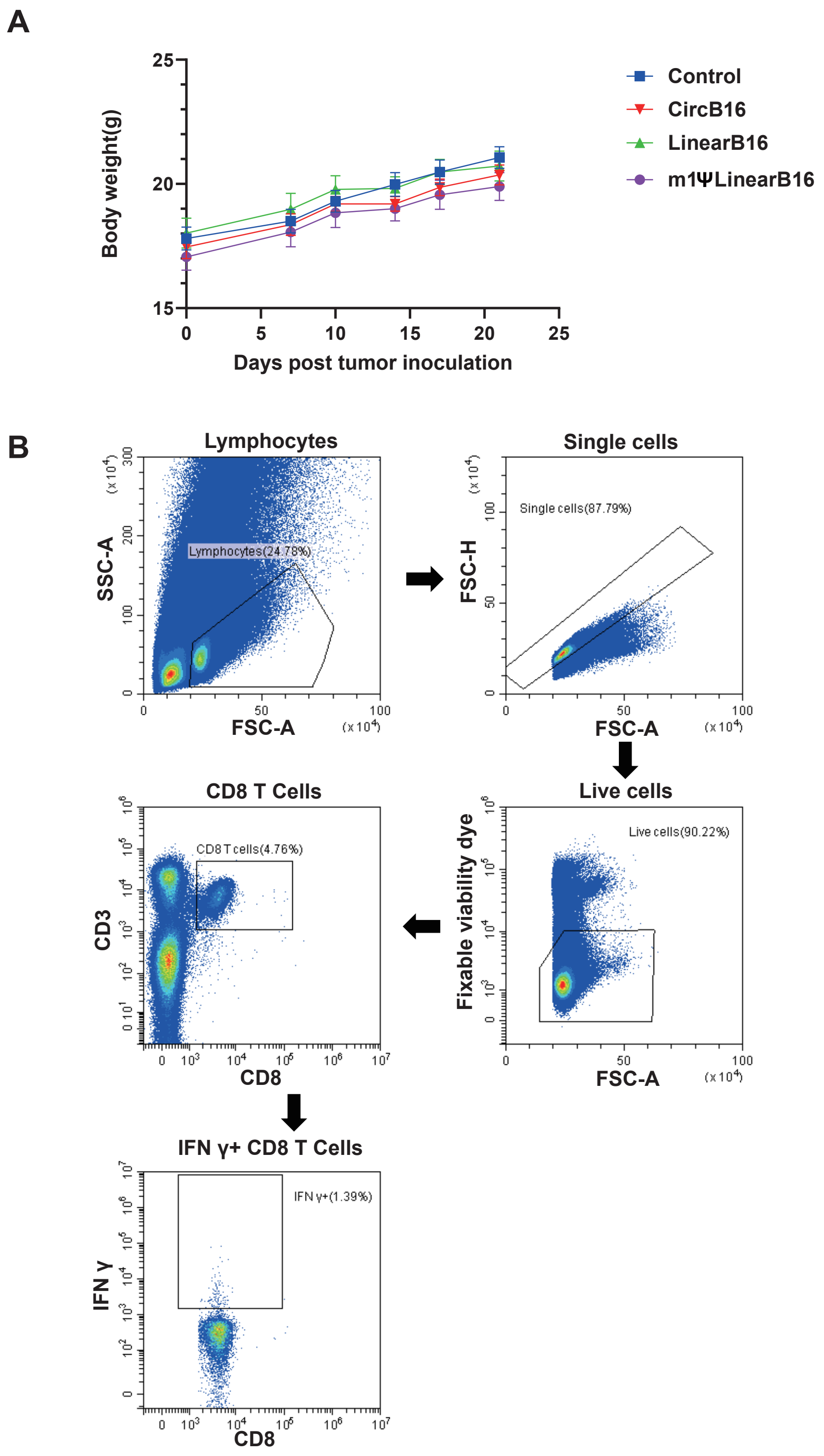

Figure S5. Body weight and gating strategy of Figure 6.

(A) Average body weight of the mice in B16F10 model. (B) Gating strategy of the flow cytometry data for detecting the percentage of IFNγ+ CD8+ T cells in Figure 6C.

Figure S6

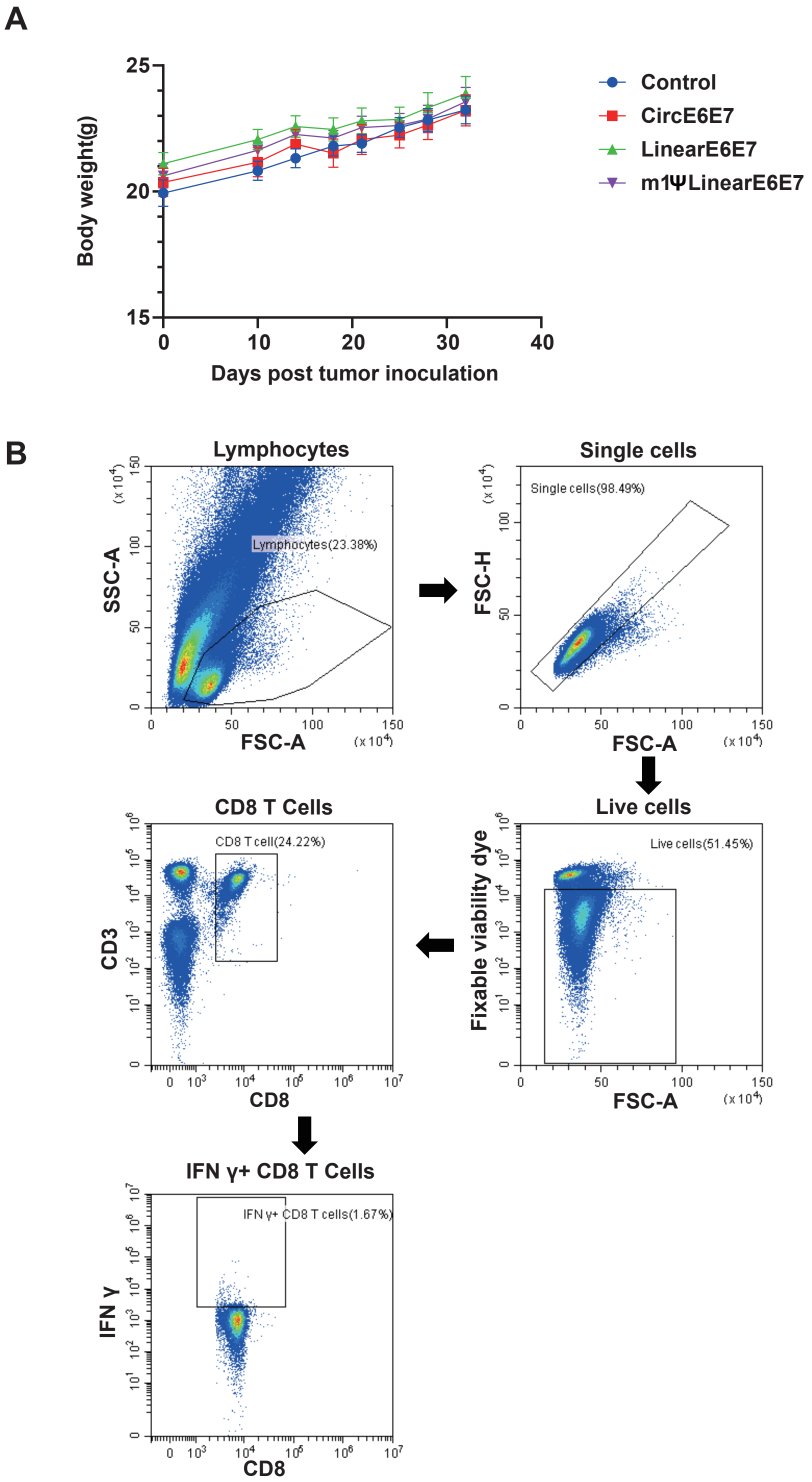

Figure S6. Body weight and gating strategy of Figure 7. (A) Average body weight of the mice in TC-1 model. (B) Gating strategy of the flow cytometry data for detecting the percentage of IFNγ+ CD8+ T cells in Figure 7B.

Figure S7

A

CircOVA

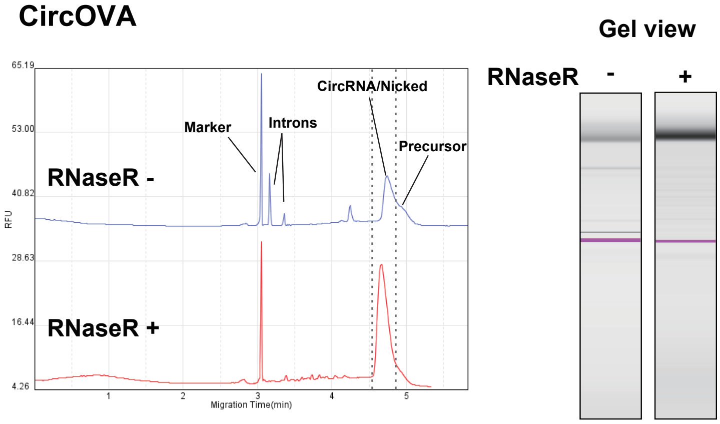

Treatment	Purity
RNaseR -	62%
RNaseR +	91.1%

B

CircB16

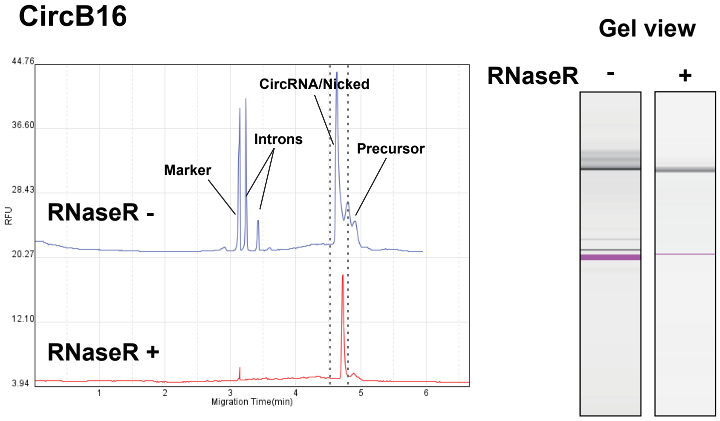

Treatment	Purity
RNaseR -	53%
RNaseR +	94.1%

C

CircE6E7

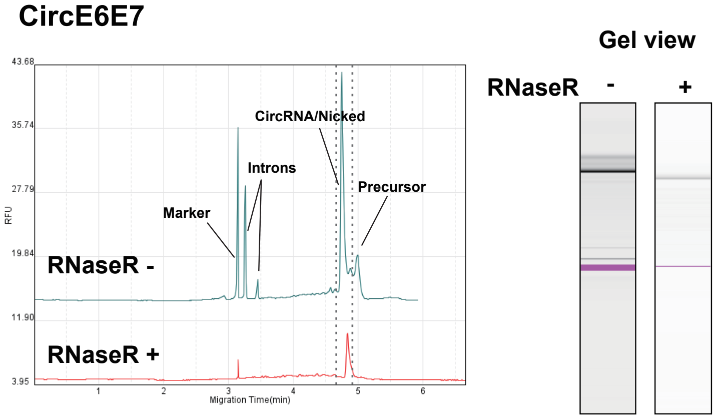

Treatment	Purity
RNaseR -	58.4%
RNaseR +	94.2%

Figure S7. Capillary electrophoresis analysis of antigen CircRNA. (A) CircOVA, (B) CircB16 and (C) CircE6E7 circRNA treated or untreated by RNaseR were analyzed by Qsep100 (BIOptic). Left panel: RFU (Relative Fluorescence Units) plots. Middle panel: Gel view. Right panel: circRNA purity percentage, CircRNA purity was calculated by circRNA area dividing total peak area.
